# Green surfactants reveal mechanistic principles governing polymeric microbubble formation and function

**DOI:** 10.1039/d6gc02897g

**Published:** 2026-08-28

**Authors:** Roman A. Barmin, Sabina A. Saraolu, Rustam A. Gumerov, Yanchen Li, MirJavad Moosavifar, Jens Köhler, Michael Pohl, Céline Bastard, Stephan Rütten, Laura De Laporte, Katharina Landfester, Igor I. Potemkin, Fabian Kiessling, Twan Lammers, Roger M. Pallares

**Affiliations:** a Institute for Experimental Molecular Imaging, RWTH Aachen University Hospital Aachen 52074 Germany rmoltopallar@ukaachen.de tlammers@ukaachen.de fkiessling@ukaachen.de; b Department of Bioengineering and Biotechnology, Faculty of Medical Engineering, National University of Science and Technology POLITEHNICA Bucharest 060042 Bucharest Romania; c Center for Molecular and Cellular Bioengineering, TUD Dresden University of Technology Dresden 01307 Germany; d DWI – Leibniz Institute for Interactive Materials Aachen 52074 Germany; e Institute of Technical and Macromolecular Chemistry, RWTH Aachen University Aachen 52074 Germany; f Department of Advanced Materials for Biomedicine, Institute of Applied Medical Engineering, RWTH Aachen University Aachen 52074 Germany; g Electron Microscope Facility, RWTH Aachen University Hospital Aachen 52074 Germany; h Max Planck Institute for Polymer Research Mainz 55128 Germany

## Abstract

Poly(butyl cyanoacrylate) microbubbles (PBCA MB) are clinically investigated ultrasound-responsive materials whose formation is governed by surfactant-mediated interfacial templating. Here, we used a chemically diverse panel of 14 commercially designated greener non-ionic surfactants to clarify how surfactant chemistry governs PBCA MB formation and performance. By combining physicochemical analysis, dissipative particle dynamics simulations, microscopy, drug-loading experiments, and acoustic characterization, we determined how the architecture of the hydrophobic moiety, PEG chain length, and hydrophilic–lipophilic balance shape MB yield, shell composition, morphology, and function. The hydrophobic tail structure primarily influenced MB yield, while increasing PEG chain length shifted the morphology toward asymmetric particles. Surfactant incorporation into PBCA MB shells depended on surfactant hydrophilic–lipophilic balance, and dissipative particle dynamics simulations indicated preferential surfactant enrichment at the polymer–water interface. Brij O20 and Ecosurf SA-9 increased drug loading by 6.2- and 4.8-fold relative to conventional Triton X-100-generated MB, while acoustic performance could be tuned toward either greater stability or stronger non-linear, disruption-prone behavior. Together, these findings clarify the role of surfactant chemistry in PBCA MB synthesis and promote surfactant design as a practical route to tailor MB morphology, shell composition, drug loading, and acoustic performance.

## Abstract 2

Greener non-ionic surfactants replace Triton X-100 in polymeric microbubble synthesis, revealing how hydrophobic tail structure, PEG length, and HLB engineer microbubble yield, morphology, shell composition, drug loading, and acoustic performance.

## Graphical abstract

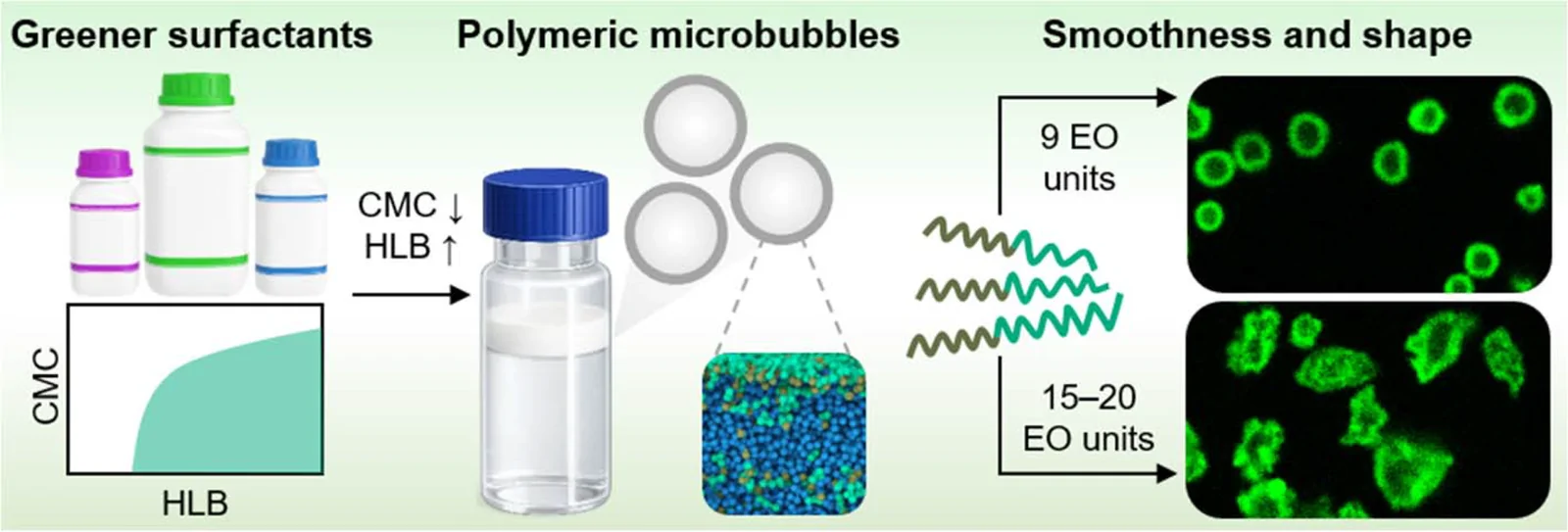

**Green foundation**

1. This work advances green chemistry by employing greener non-ionic surfactants compared to Triton X-100 to elucidate structure–property relationships in polymeric microbubble synthesis.

2. Fourteen greener surfactants were screened, of which six supported microbubble formation. Brij O20 and Ecosurf SA-9 significantly enhanced drug loading, while acoustic performance could be tuned toward either greater stability or stronger non-linear, disruption-prone behavior.

3. These findings establish a framework for surfactant engineering in polymeric microbubble synthesis, supporting microbubble design towards specific applications.

## 1. Introduction

Alkylphenol ethoxylates are non-ionic surfactants widely used in industrial formulations and laboratory applications, with Triton X-100 being one of their most prominent representatives.^1,2^ Due to their high performance, chemical stability, and cost-effectiveness, these compounds have been adopted in manufacturing processes worldwide.^3,4^ However, they degrade into alkylphenols, bioaccumulative and environmentally persistent compounds with documented ecotoxic effects, particularly in aquatic environments.^5,6^ Alkylphenols have been shown to disrupt hormonal systems in wildlife and reduce reproductive fitness in organisms.^7–9^ As a result, the European Union has progressively restricted the use of alkylphenol ethoxylates, listing them for authorization and imposing concentration limits in certain applications, while promoting the transition toward safer and more sustainable surfactant alternatives.^10,11^

Although current regulatory restrictions do not specifically target nano- and microscale (bio)materials aimed for clinical use, Triton X-100 remains widely involved in their synthesis.^12–14^ In polymeric microcapsule synthesis, particularly *via* (mini)emulsion polymerization, Triton X-100 can act as a non-ionic emulsifier that reduces interfacial tension and stabilizes dispersed droplets or gas bubbles against coalescence.^15,16^ These structures serve as confined reaction sites where polymerization proceeds, forming a polymer shell around a liquid or gaseous core.^17,18^ Among these systems, gas-filled microcapsules (so-called microbubbles, MB) based on poly(butyl cyanoacrylate) (PBCA) are particularly notable for their intrinsic acoustic responsiveness, high drug loading capacity, and favorable biocompatibility profile, as cyanoacrylate-based materials are approved by the American Food and Drugs Administration for medical adhesive applications.^19,20^ PBCA MB were initially introduced for clinical ultrasound (US) imaging and are now emerging as therapeutic enhancers, enabling transient and localized drug delivery upon US activation across biological barriers, for example in the brain or tumor vasculature.^21,22^

Recent advances in PBCA MB engineering have distinctly improved both their diagnostic sensitivity and therapeutic performance.^23–27^ In particular, controlling interfacial templating during MB synthesis offers a valuable opportunity to tailor MB performance, although the key surfactant properties governing this process remain unidentified. In a recent study, we addressed this by screening surfactants from the Tween and Triton X families, showing that increasing surfactant molecular weight (MW) led to MB with thicker shells and altered acoustic properties; however, it remained challenging to disentangle the specific compositional features responsible for these effects.^28^ The rapid development of greener alternatives to Triton X-100 therefore offers an opportunity to systematically identify the surfactant characteristics governing MB performance while simultaneously improving the sustainability of the synthesis process.^29^

Here we systematically evaluate 14 commercially designated greener non-ionic surfactants to clarify how surfactant chemistry governs PBCA MB synthesis and performance. Combining physicochemical analysis, dissipative particle dynamics simulations, microscopy, drug-loading experiments, and acoustic characterization, we determine how the architecture of the hydrophobic moiety, the hydrophilic PEG chain length, and the hydrophilic–lipophilic balance (HLB) shape MB yield, shell composition, morphology, and performance. In this way, the present work aims to move from empirical surfactant selection toward design principles for application-oriented PBCA MB engineering.

## 2. Results and discussion

### 2.1 Greener surfactant selection for polymeric microbubble synthesis

**Fig. 1:**
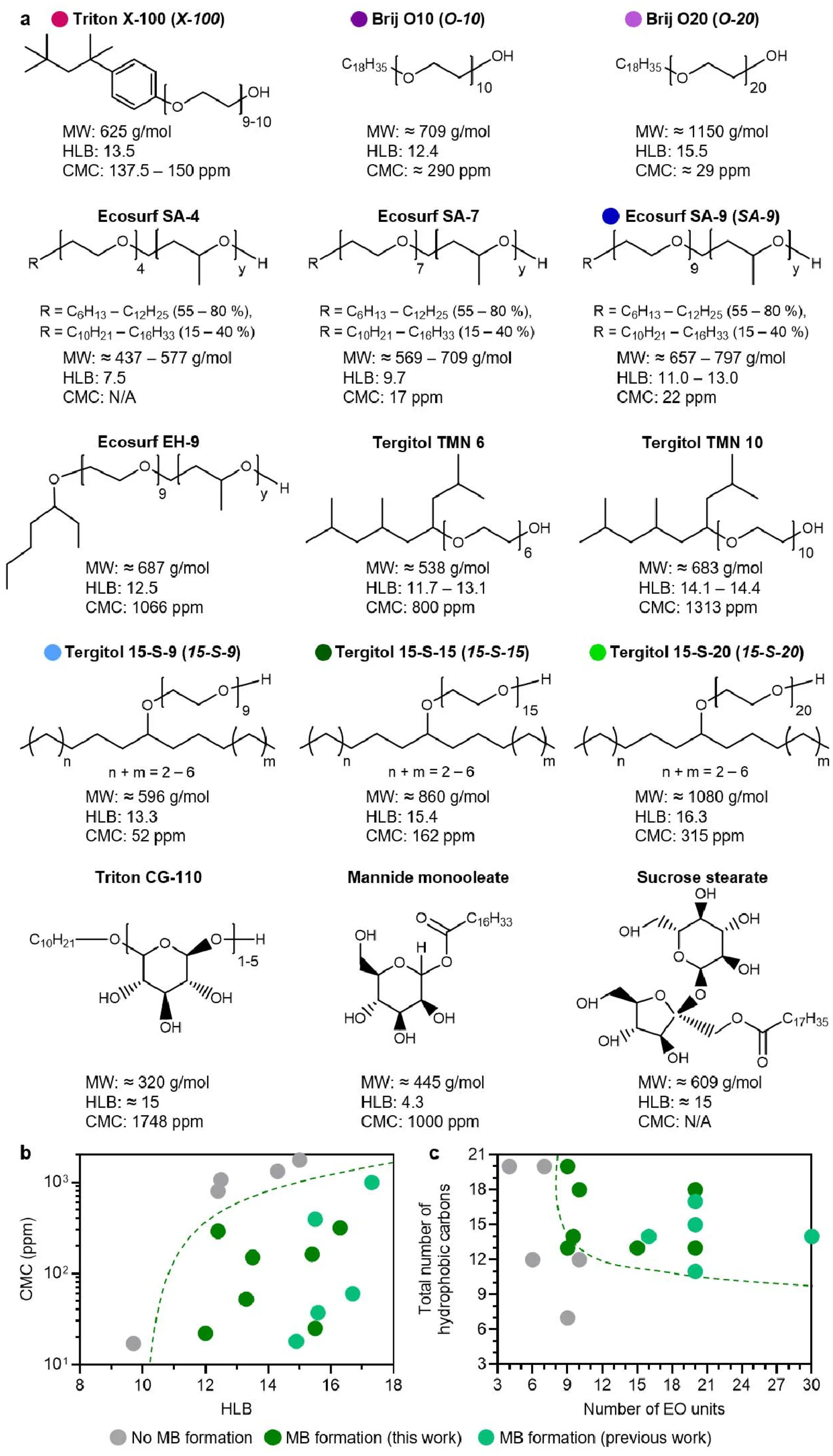
Greener surfactant selection for PBCA MB synthesis. (a) Chemical structures of the surfactants together with their molecular weights (MW), hydrophilic–lipophilic balance (HLB) values, and critical micelle concentrations (CMC). Colored spheres indicate surfactants that yielded successful PBCA MB synthesis, with colors corresponding to the coding used throughout the manuscript. (b) Correlation between surfactant HLB and CMC and their ability to promote MB formation observed in this and our previous works.^28^ (c) Correlation between PEG-based surfactant EO unit number and total hydrophobic carbon number and the ability to promote MB formation. Surfactant concentrations and volumes used for PBCA MB synthesis and storage are provided in Table S1, while surfactant solubility and its influence on MB synthesis and retrieval are summarized in Table S2. Green dashed lines illustrate the hypothesized boundary between successful and unsuccessful MB formation.

Fig. 1a shows the chemical structures of the green surfactants investigated in this study, together with their MW, HLB values, and critical micelle concentrations (CMC), when reported by the manufacturer. The inclusion criteria were low-cost and explicit designation by the manufacturer as Greener Alternative Products aligned with the 12 Principles of Green Chemistry.^30^ All selected surfactants were non-ionic, as Triton X-100 belongs to this class. Non-ionic surfactants have been reported to produce smaller bubble templates than charged surfactant,^31^ while cationic surfactants are generally associated with higher toxicity than other surfactant classes.^32^ Accordingly, the greener surfactants investigated here fall into two broad classes: non-sugar-based and sugar-based non-ionic surfactants. The following paragraphs discuss these classes separately.

Four surfactant families (Brij, Ecosurf, Tergitol TMN, and Tergitol 15-S) were selected as potential non-sugar-based alternatives to Triton X-100, enabling a systematic evaluation of how their chemical structures, including variations in hydrophobic tails and hydrophilic poly(ethylene glycol) (PEG) content, influence MB synthesis and properties. The Brij O series consists of non-ionic surfactants based on ethoxylated oleyl alcohols.^33,34^ Brij O10 and Brij O20 possess the same linear oleyl hydrophobic moiety but differ in PEG chain length (10 *vs.* 20 ethylene oxide (EO) units). The PEG length of Brij O10 is comparable to that of Triton X-100; consequently, the surfactants differ in HLB values. The Ecosurf SA family comprises ethoxylated propoxylated alcohols.^35^ Members SA-9, SA-7, and SA-4 were selected to systematically decrease the PEG content from 9 EO units (comparable to the PEG segment of Triton X-100) to 4 EO units while maintaining similar linear hydrophobic tail mixtures. In addition, Ecosurf EH-9 was included to investigate how modifying the hydrophobic domain from linear to branched, while keeping the PEG chain length constant, affects MB formation. Unlike the other surfactants investigated here, where hydrophobic tails are directly linked to hydrophilic units, Ecosurf surfactants contain a short propylene oxide (PO) segment that separates and shields the PEG chains; these units are more hydrophobic than their EO counterparts. The Tergitol series consists of branched secondary alcohol ethoxylates, of which two families were considered.^36^ The TMN series contains a trimethylnonyl hydrophobic moiety; TMN-10 contains approximately 10 EO units, comparable to Triton X-100, whereas TMN-6 contains six EO units. The 15-S series consists of mixtures of secondary ethoxylated alcohols, with PEG chain lengths of 9, 15, and 20 EO units for 15-S-9, 15-S-15, and 15-S-20, respectively. Across all four surfactant families, increasing the number of EO units while maintaining the same hydrophobic moiety leads to higher HLB and CMC values, consistent with the reported behaviors for non-ionic ethoxylated surfactants.^37^

Triton CG-110, sucrose stearate, and mannide monooleate were selected as sugar-based surfactants derived from renewable carbohydrate feedstocks and fatty acids.^38^ Their sugar-based headgroups provide a renewable and hydrophilic motif that can support effective interfacial stabilization. Triton CG-110 (decyl glucoside) belongs to the alkyl polyglucoside family and consists of a linear decyl hydrophobic tail linked to a glucose-derived hydrophilic headgroup, designed to replace Triton X-100 in biopharmaceutical applications.^39,40^ Sucrose stearate is a sucrose fatty acid ester formed by esterification of sucrose with stearic acid, resulting in a hydrophobic C_18_ alkyl chain attached to a carbohydrate headgroup.^41^ Mannide monooleate is produced from mannide (a dehydrated sugar alcohol related to sorbitol) and oleic acid, forming an amphiphilic structure with a sugar-based hydrophilic core and a C_18_ unsaturated hydrophobic chain.^42^

Prior to PBCA MB synthesis, stock synthesis and storage solutions were prepared as listed in Table S1, maintaining surfactant molar concentrations comparable to those used for Triton X-100.^43,44^ The storage solutions were adjusted to pH 7.0, and all surfactants dissolved at 0.3 mM, as shown in Table S2. The synthesis solutions were adjusted to pH 2.5 to ensure a slow and controllable polymerization of the highly reactive butyl cyanoacrylate monomers during high-speed stirring, allowing the growing polymer chains to form the MB shells.^15,16^ As indicated in Table S2, all non-sugar-based surfactants were readily dissolved in solutions adjusted to pH 2.5, as was the sugar-based Triton CG-110. In contrast, the other two sugar-based surfactants, namely mannide monooleate and sucrose stearate, remained insoluble under these conditions. This behavior can be attributed to their limited aqueous solubility influenced by their long hydrophobic fatty acid chains and fewer strongly hydrophilic groups.^38^ Mannide monooleate additionally formed a precipitate, most likely due to acid-catalyzed hydrolysis of its ester linkage, releasing oleic acid.^45^

To assess the relationship between surfactant physicochemical properties and their ability to promote MB formation, we analyzed the HLB and CMC values of surfactants that were soluble under the MB synthesis conditions. HLB and CMC are key physicochemical parameters governing surfactant (self-)assembly and colloidal stability: HLB helps determine emulsion type, while CMC defines the concentration threshold for micelle formation and dilution stability.^46,47^ To broaden the dataset, we additionally incorporated the CMC–HLB profiles of five Triton X and Tween surfactants examined in our previous work.^28^ As shown in Fig. 1b, successful MB formation clustered within a region defined by HLB values above 10 and CMC values in the range of 10–1000 ppm. We emphasize that neither parameter directly describes adsorption or surface tension at the air–water interface, and the observed HLB–CMC region should therefore be regarded as an empirical physicochemical window associated with successful MB formation rather than evidence for a specific interfacial adsorption mechanism. Air–water interfacial properties, including surface tension and surfactant adsorption behavior, are also expected to contribute to stabilization of the initially formed gas bubbles. In our previous study employing Triton X and Tween surfactants, synthesis solutions showed air–liquid interfacial tensions within a relatively narrow range (28–42 mN m^−1^), despite differences in the resulting MB properties.^28^ Accordingly, surface tension was not included among the primary physicochemical descriptors investigated in the present surfactant screening. HLB was retained as a descriptor of surfactant amphiphilic balance because MB synthesis subsequently involves formation of a hydrophobic PBCA phase.

To gain further structural insight into the effects of non-sugar-based surfactants on MB synthesis, we analyzed the relationship between the number of EO units, representing the hydrophilic portion of the surfactant, and the total number of hydrophobic carbons, used as a model descriptor of the hydrophobic moiety of each compound. For surfactants with mixed hydrophobic tails, average numbers of hydrophobic carbons were used. Fig. 1c suggests that surfactants with 9 or more EO units are required for MB formation; however, the hydrophobic moiety must adequately counterbalance the hydrophilicity imparted by the PEG chain, consistent with the need to maintain an appropriate amphiphilic balance for interfacial stabilization.

### 2.2 Greener surfactant chemistry tunes the physicochemical profile and shell composition of polymeric microbubbles

**Fig. 2:**
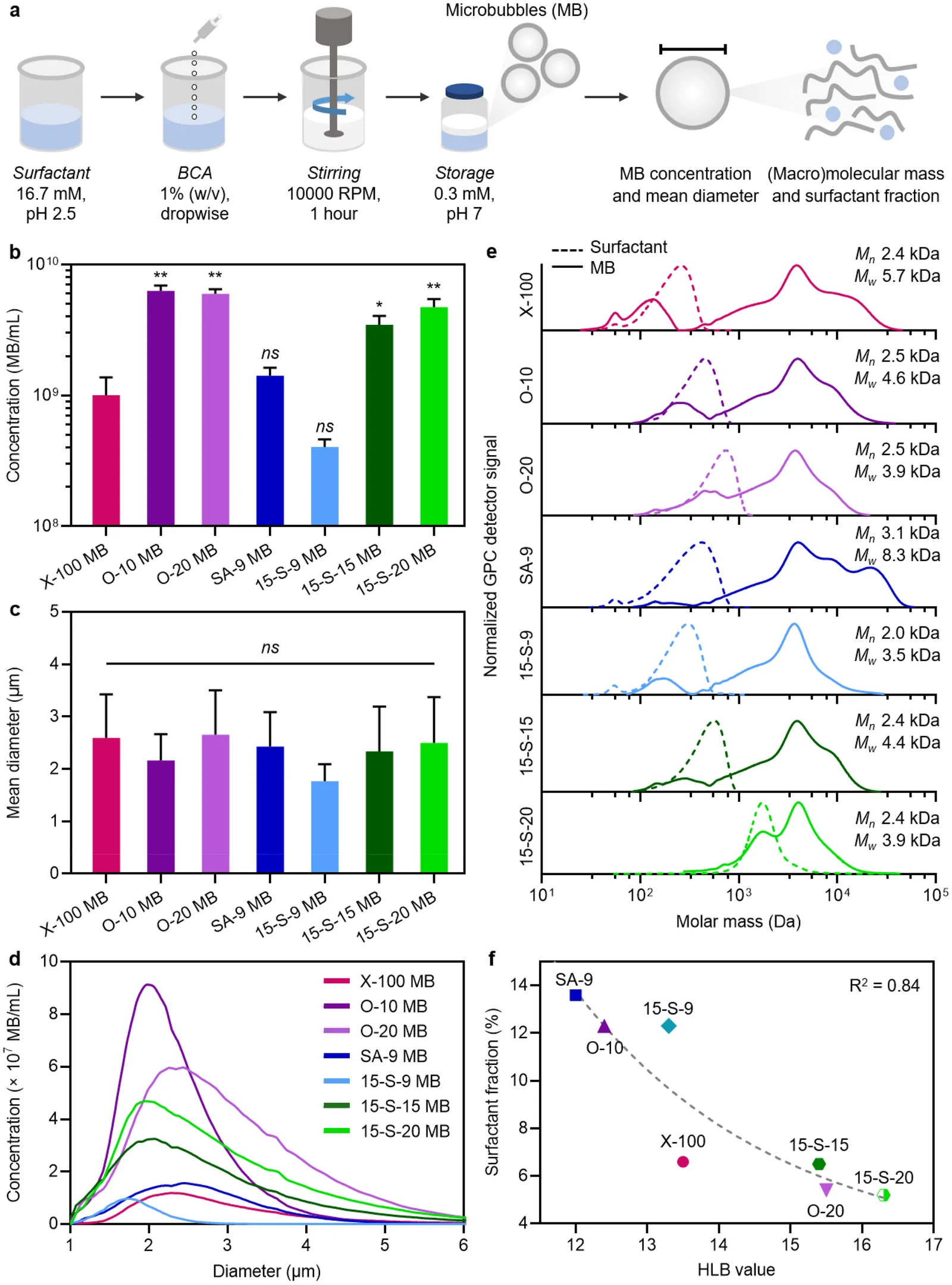
Greener surfactant composition tunes the physicochemical profile of PBCA MB. (a) Each MB sample was synthesized using an equal surfactant concentration (16.7 mM) by anionic polymerization of BCA under high-speed stirring. After purification, all samples were stored in a solution containing the corresponding surfactant (0.3 mM), and both intact MB and lyophilized polymer powders were characterized. (b) MB concentration, (c) mean diameter, and (d) diameter distribution profiles obtained *via* Coulter counter measurements. (e) Molecular mass distribution profiles obtained from size-exclusion chromatography (SEC), and (f) surfactant fractions incorporated onto and into the MB shell as a function of parent surfactant HLB. Values represent mean ± standard deviation of three different batches of PBCA MB, each measured in triplicates. (*) and (**) indicate groups that are significantly different with *p* < 0.05 and *p* < 0.01, respectively; (ns) indicates groups that are not significantly different with *p* > 0.05 (one-way ANOVA with *post hoc* Tukey HSD test). PBCA MB concentration and mean diameter values are listed in Table S3.

Fig. 2a shows a schematic representation of PBCA MB synthesis. After preparation of the synthesis solution, 1% (w/v) butyl cyanoacrylate was added dropwise under high-speed stirring, generating surfactant-stabilized gas bubbles that served as templates for MB formation. Under the acidic synthesis conditions, monomer underwent anionic polymerization, and the growing PBCA chains progressively reinforced the bubble interface to form the polymeric MB shell, being consistent with our reported protocols.^26,28^ As shown in Fig. 1 and Table S2, only 6 of the 14 Triton X-100 greener alternatives resulted in MB formation. Those include Brij O10 and Brij O20 (referred to as O-10 MB and O-20 MB, respectively), Ecosurf SA-9 (labeled SA-9 MB), Tergitol 15-S-9, 15-S-15, and 15-S-20 (referred to as 15-S-9 MB, 15-S-15 MB, and 15-S-20 MB, respectively). For consistency, PBCA MB synthesized with Triton X-100 are referred to as X-100 MB.

Regarding the surfactants that failed to produce MB, several factors were identified as potentially contributing to unsuccessful syntheses. For instance, while Ecosurf SA-9 yielded stable PBCA MB, its analogues with shorter PEG chains (Ecosurf SA-7 and SA-4) resulted primarily in foam formation, likely due to their lower HLB and increased hydrophobicity (reflecting trends observed in Fig. 1b and c, respectively), which reduces aqueous solubility and alters interfacial packing at the gas–liquid interface. In contrast, Ecosurf EH-9 also failed to produce PBCA MB, likely because its shorter (although branched) hydrophobic moiety reduces interfacial anchoring compared with Ecosurf SA-9, limiting stabilization of the forming polymer shell. Its much higher CMC also places it outside the CMC range associated with successful MB formation in the present surfactant panel. While Triton X-100 and Tergitol TMN 10 contain comparable numbers of PEG units, the inability of Tergitol TMN 10 to produce PBCA MB may stem from the weaker interfacial anchoring of its aliphatic trimethylnonyl part compared with the aromatic octylphenyl moiety of Triton X-100. TMN 6 was likewise ineffective in MB formation. In addition, both TMN surfactants exhibit relatively high CMC values and fall outside the empirical CMC range associated with successful MB formation. Similarly, Triton CG-110, which exhibits a relatively high CMC together with a strongly hydrophilic glucose headgroup, failed to form PBCA MB.

Following synthesis and purification, the MB were transferred to a storage solution and analyzed using a Coulter Counter to determine MB concentration and mean diameter. Fig. 2b shows the concentration profiles of the synthesized MB. O-10 MB exhibited the highest concentration among all samples, showing a 6.2-fold increase compared with conventional X-100 MB. While both surfactants contain comparable numbers of EO units within their PEG chains, the long, flexible oleyl chain of Brij O10 likely provides more efficient stabilization of the gas–liquid interface than the bulkier and more rigid octylphenyl hydrophobic moiety of Triton X-100. Further increasing the number of EO units up to 20 did not significantly compromise the MB formation capacity of the Brij surfactants, with O-20 producing a 5.9-fold higher MB yield than its X-100 MB counterpart. 15-S-9 MB showed a 2.5-fold lower concentration compared with X-100 MB, despite having equal numbers of EO units. Further increasing its number from 9 to 15 and 20 within the Tergitol 15-S family resulted in 3.4- and 4.7-fold higher concentrations than X-100 MB for 15-S-15 MB and 15-S-20 MB, respectively. The lower yield of 15-S-9 MB may arise from its C_11_–C_15_ secondary alcohol hydrophobic tail mixture, which lacks both the aromatic interfacial anchoring of Triton X-100 and the long, uniform oleyl chain of Brij O10, leading to less efficient packing and stabilization at the gas–liquid interface. Although Ecosurf SA-9 contains a linear hydrophobic tail (similar to Brij O10) and a PEG length comparable to Triton X-100, the PO block adjacent to the PEG chain likely modifies the interfacial packing of the surfactant, leading to SA-9 MB concentrations comparable to those of X-100 MB. These trends are consistent with efforts to optimize hard-shelled MB yield, where even minor modifications to the synthesis, such as introducing small molecules or copolymers, can noticeably affect MB concentration profile.^48,49^

Although the mean diameters of all synthesized MB were not statistically different and remained ∼2 µm (Fig. 2c), diameter profiles shown in Fig. 2d reveal a slight increase in MB size with increasing number of EO units within both the Brij O and Tergitol 15-S families. This is consistent with our previous results obtained with PBCA MB synthesized with Triton X series.^28^ We propose that surfactants define the colloidal templating of gas bubbles, which are initially stabilized by surfactant shells and subsequently reinforced by PBCA polymerization, analogous to other dispersed surfactant-stabilized systems.^17,50^ Accordingly, the balance between hydrophobic and hydrophilic motifs can tune the resulting PBCA MB yield and diameter. Based on these results and our previous studies on MB synthesized with Triton X and Tween surfactants,^28^ we hypothesize that the hydrophobic moiety, particularly its composition and flexibility, primarily governs PBCA MB yield, whereas the length of the hydrophilic (*e.g.* PEG) segment modulates the MB diameter distribution.

Molecular mass and molecular mass distribution of the polymer chains of the PBCA MB were analyzed using size-exclusion chromatography (SEC). The composition was further analyzed *via* proton nuclear magnetic resonance (NMR) spectroscopy. For SEC and NMR analyses, polymeric MB were washed with deionized (DI) water to remove residual surfactant, freeze-dried, and the resulting polymer powders were dissolved in chloroform. The SEC molar mass distribution profiles indicate that all MB samples consist of polymer chains with number-average (*M*_n_) and weight-average (*M*_w_) molar masses below 40 kDa (Fig. 2e), being consistent with our previous reports.^28,43^ These relatively low molecular weights may enable faster hydrolytic degradation after MB uptake by the liver and spleen.^51,52^ Low-molecular-weight signals between 300 Da and 3 kDa indicate the presence of surfactant residues in the MB shells and are comparable to those signals observed for the surfactants alone. Furthermore, the SEC chromatograms of all MB samples showed a main signal between 2 and 6 kDa, while most samples exhibited signals with low intensity between 8 and 12 kDa, agreeing with previous reports on cyanoacrylate polymerization in the presence of colloidal templates.^53,54^*M*_n_ values (shown in Fig. 2e) were low across all MB samples (around 2.0–3.1 kDa), indicating generally short polymer chains. To better reflect the presence of higher molecular weight fractions and the overall polydispersity, *M*_w_ values are also provided. Notably, SA-9 MB exhibited a higher *M*_w_, indicating the retention of longer chains and a broader distribution. This may be related to the PO-containing architecture of the parent Ecosurf SA-9 surfactant, which differs from the more conventional amphiphiles used here and may promote a less compact, more heterogeneous interfacial layer during template polymerization.^55,56^

The NMR spectra of the parent surfactants and the corresponding PBCA MB samples were recorded (Fig. S1–S7). For each surfactant, complete peak assignments are provided according to the chemical structures shown in Fig. 1. The presence of surfactant residues was confirmed by peaks originating from the surfactants in the spectra of the PBCA chains. According to NMR analysis, surfactant residues accounted for 5.2–13.6% of the MB shell signal, as shown in Fig. 2f. Notably, the data suggest a possible exponential dependence of surfactant incorporation into the MB shell on the HLB of the parent surfactant. Although the fit yielded an *R*^2^ of only 0.84, this exploratory trend may provide a useful rule of thumb for surfactant selection in MB synthesis. For example, while Ecosurf SA-9 successfully yielded PBCA MB, the lower-HLB surfactants SA-7 and SA-4 would be predicted to require surfactant fractions of 53% and 26.8% in the MB shell, respectively, which is likely too high to enable effective gas–liquid interface stabilization. In contrast, Ecosurf EH-9, Tergitol TMN 6, and Tergitol TMN 10 would be predicted to require only 12.0%, 14.1%, and 7.7% surfactant incorporation, respectively; however, all three exhibit relatively high CMC values and fall outside the empirical CMC range associated with successful MB formation. Since CMC describes bulk self-assembly rather than interfacial adsorption, this observation alone does not identify the mechanism underlying their inability to form MB.

**Fig. 3:**
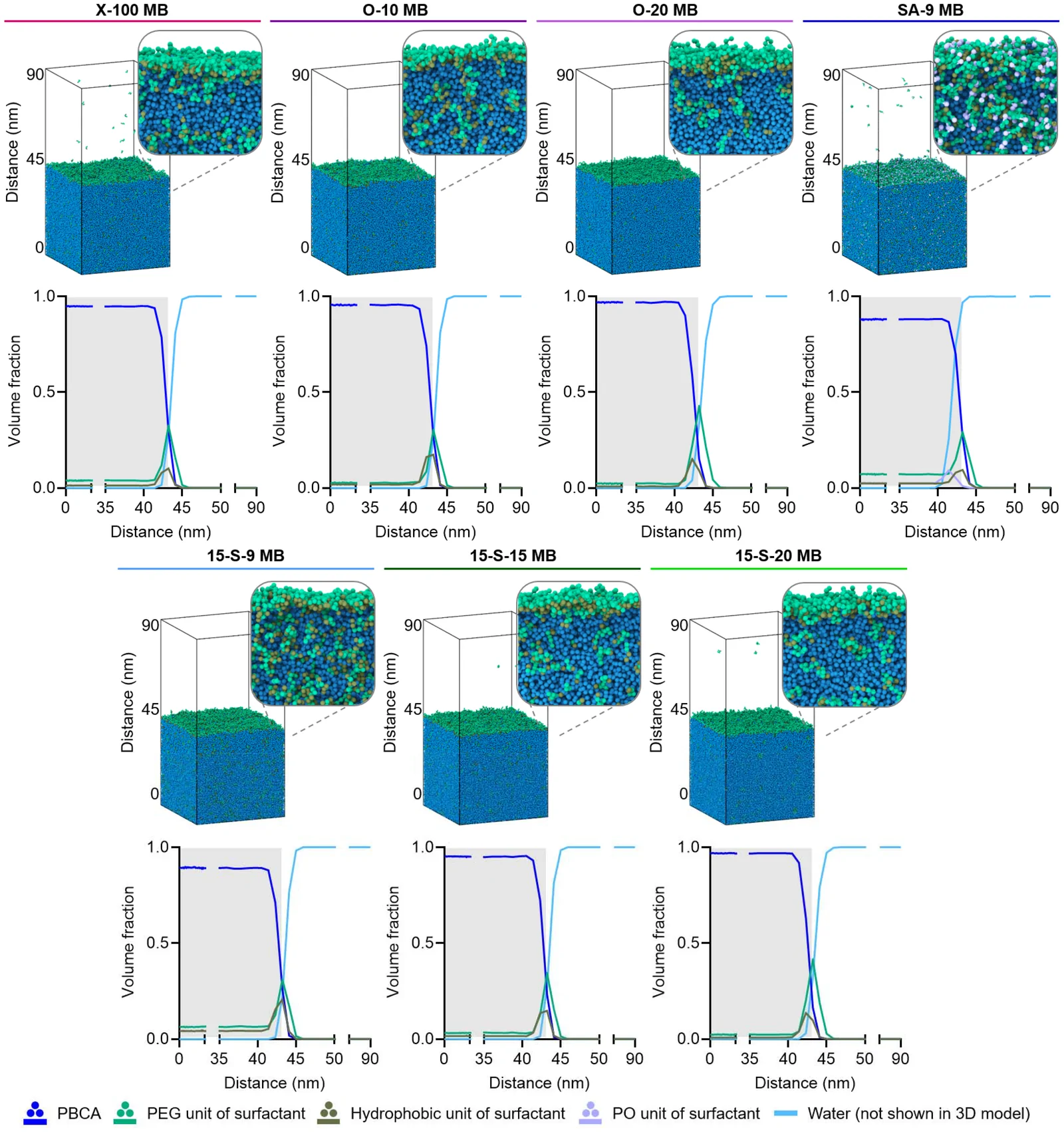
DPD simulations of surfactant-based PBCA MB shells, including simulation snapshots of the polymeric shells near the water–shell interface and corresponding density profiles of MB shell fragments. In the simulation snapshots, the water molecules are omitted for clarity (transparent regions above the polymeric shells), while they were considered during the simulation hence, shown in the density profiles. The gray background in the density profiles represents the MB shell. Hansen solubility parameters, structural formulas of the coarse-grained surfactant models, and the DPD interaction parameters used in the simulations are listed in Tables S4–S6, respectively.

To further elucidate the role of surfactants in MB synthesis, dissipative particle dynamics (DPD) simulations were performed to model MB shell stabilization in the presence of different architectures. Fig. 3 shows the internal structures of the simulated MB shells, together with density profiles plotted along the direction normal to the MB surface. The total surfactant concentration in the modeled box section was set to the experimentally determined surfactant fraction values shown in Fig. 2f. The simulations showed an inhomogeneous distribution of surfactant molecules within the PBCA MB shells, with a maximum concentration at the polymer–water interface, being consistent with our previous report.^25^ The relatively hydrophobic nature of PBCA promotes surfactant accumulation at the shell–water interface to reduce interfacial free energy, with the hydrophilic units oriented toward the aqueous phase.^57^ The mean surfactant concentration inside the shell was slightly lower than the overall surfactant concentration, correlating with the HLB of the surfactant. For example, in MB shells containing Tergitol 15-S-20, which had the highest HLB, experimentally measured surfactant concentration was 5.2%, while the modeled surfactant concentration inside the shell was 3.1%. In contrast, for Ecosurf SA-9, which had the lowest HLB value, the corresponding experimentally measured and modeled shell-interior surfactant concentrations were 13.6%, and 12.0% respectively.

### 2.3 Greener surfactants impact microbubble morphology, sphericity, and shell thickness

**Fig. 4:**
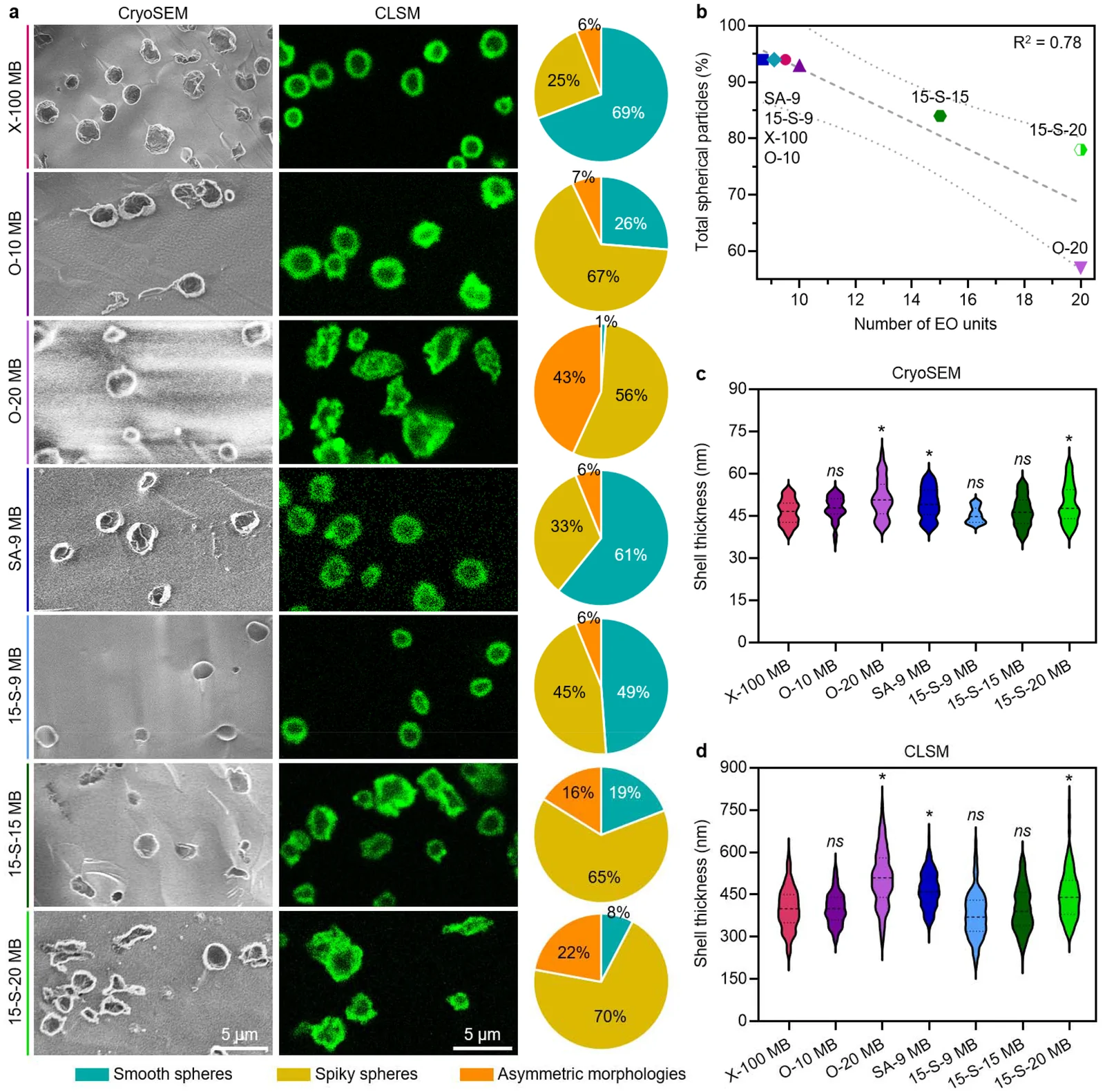
Morphological analysis of surfactant-based PBCA MB. (a) Representative cryoSEM, and CLSM micrographs of MB, together with pie charts showing the distribution of particle morphologies based on CLSM images, including smooth spheres, spiky spheres, and asymmetric morphologies. (b) Correlation between surfactant EO number and the total spherical particle fraction. Gray dashed lines indicate the linear fit and its 95% confidence interval. Shell thickness measurements of PBCA MB by (c) cryoSEM, and (d) CLSM images. (*) indicates groups that are significantly different with *p* < 0.05; (ns) indicates groups that are not significantly different with *p* > 0.05 (one-way ANOVA with *post hoc* Tukey HSD test).

To assess MB morphology, two complementary imaging modalities were employed. Plain MB were imaged by scanning electron cryo-microscopy (cryoSEM), whereas MB loaded with the fluorescent dye were analyzed by confocal laser scanning microscopy (CLSM). Representative images are shown in Fig. 4a. Both imaging modalities showed that surfactants with a PEG unit length of 9–10 predominantly yielded spherical (smooth or spiky) MB, whereas increasing the PEG unit length to 20 resulted in a higher fraction of particles with non-spherical asymmetric morphologies with circularity below 0.8. Because CLSM and cryoSEM characterize MB in different states, CLSM micrographs were used for the sphericity analysis. Hence, whereas CLSM images MB in their native hydrated environment, cryoSEM analyzes dehydrated samples and may therefore underestimate particle sphericity profile. Based on the CLSM images, particles were classified into three main morphological categories: smooth spheres, spiky spheres, and asymmetric morphologies. Representative pie charts summarizing the shape distribution are shown in Fig. 4a. Among the samples, X-100 MB exhibited the highest fraction of smooth spheres (69%), followed by SA-9 MB (61%), 15-S-9 MB (49%), and O-10 MB (26%). In particular, Triton X-100 may promote the most uniform MB morphology because alkylphenol ethoxylates are known to form more compact interfacial monolayers than alcohol ethoxylates, whereas the oleyl-based hydrophobic moiety of Brij O-10 likely introduces greater interfacial disorder.^58^ Moreover, the fraction of asymmetric MB in these samples (X-100 and O-10 MB) did not exceed 7%, indicating that they largely retained a spherical morphology, either smooth or spiky. In contrast, O-20 MB and 15-S-20 MB exhibited the highest fractions of particles with asymmetric morphologies (43% and 22%, respectively). A plausible explanation is that increasing the number of EO units to 20 enlarges the effective hydrophilic headgroup and steric footprint of the surfactant, resulting in a more hydrated and less compact interfacial layer during template polymerization.^59,60^ This may reduce uniform interfacial stabilization of nascent MB and thereby increase the fraction of non-spherical particles.^61^ Consistent with this interpretation, the linear fit for the correlation between surfactant EO unit number and the total spherical particle fraction, considering both smooth and spiky spheres, showed a good agreement (Fig. 4b). The correlation between surfactant EO unit number and the smooth sphere fraction of PBCA MB is shown in Fig. S8 and exhibits a trend similar to that observed in Fig. 4b.

Subsequently, shell thickness was measured from cryoSEM and CLSM micrographs of PBCA MB, as shown in Fig. 4c and d, respectively. While cryoSEM analyzes dehydrated samples and may therefore slightly underestimate shell thickness,^62–64^ the lower resolution of CLSM may lead to overestimation.^65^ Nevertheless, both methods showed consistent trends between surfactant MW and MB shell thickness similar to our previous report,^28^ with the true shell thickness likely lying between the values obtained by both techniques. All samples showed comparable shell thickness values of ∼50 nm by cryoSEM (Fig. 4c), and ∼400 nm by CLSM (Fig. 4d), consistent with our previous reports.^64,66^ Although O-20 MB, SA-9 MB, and 15-S-20 MB exhibited slightly higher shell thickness values that were statistically significant, these samples also showed greater variability. We attribute this to the more pronounced morphological effects compared to the X-100 MB counterparts. In particular, the extended PEG chains (20 units in O-20 MB and 15-S-20 MB) and the PO-containing architecture of SA-9 MB, which also gave rise to greater polymer chain dispersity (Fig. 2e), promoted the formation of more spiky or non-spherical MB (Fig. 3), leading to a broader dispersion of shell thickness values.

### 2.4 Greener surfactants tune microbubble drug loading and acoustic responses

**Fig. 5:**
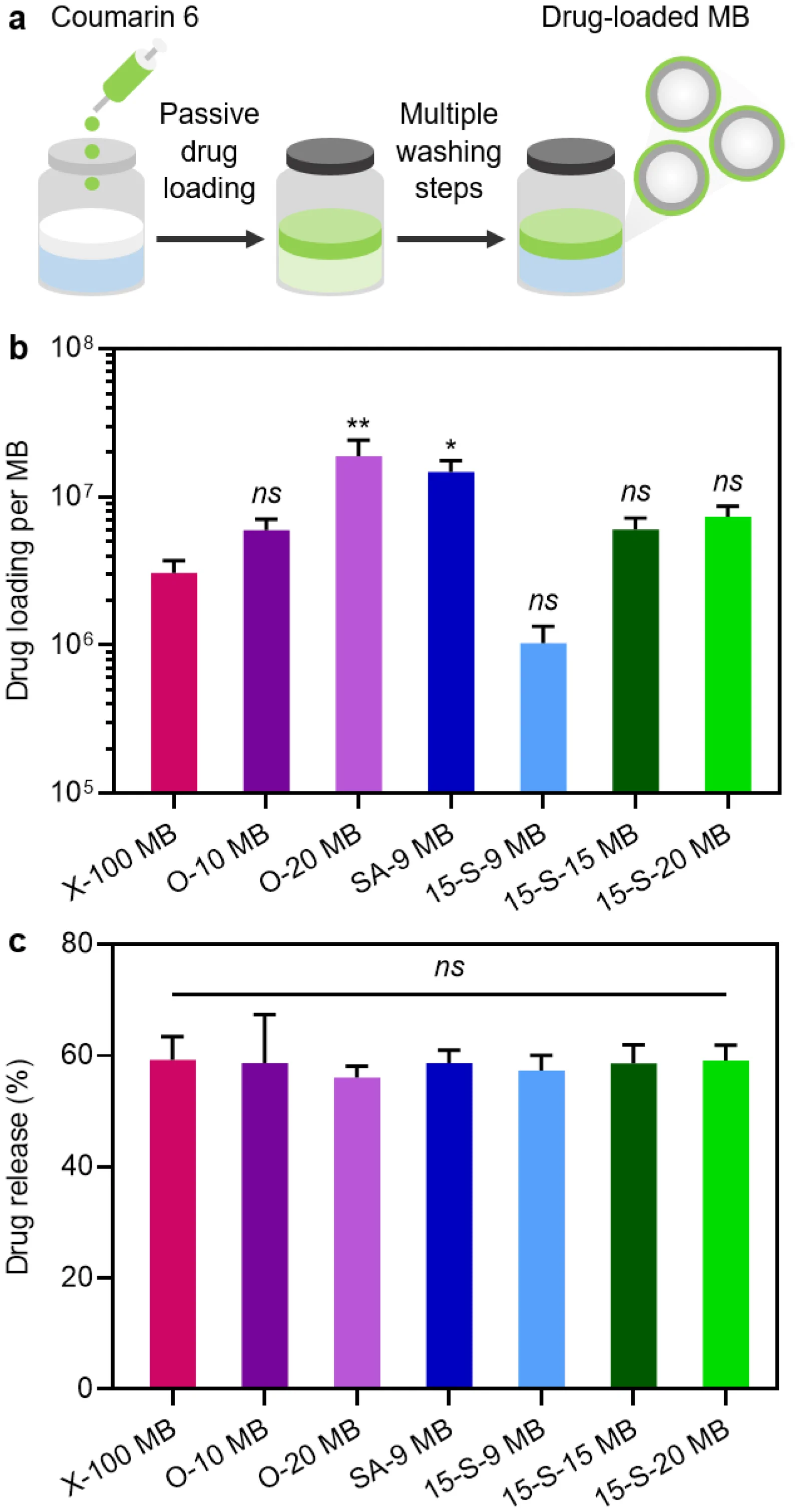
Drug loading profiles of surfactant-based PBCA MB. (a) Schematic representation of the coumarin 6 loading protocol. (b) Number of coumarin 6 molecules loaded per MB and (c) drug release percentage by the different MB samples upon US exposure. Values represent mean ± standard deviation of three different batches of PBCA MB, each measured in triplicates. (*) and (**) indicate groups that are significantly different with *p* < 0.05 and *p* < 0.01, respectively; (ns) indicates groups that are not significantly different with *p* > 0.05 (one-way ANOVA with *post hoc* Tukey HSD test). Drug loading values per individual MB and per 10^9^ MB are listed in Table S7.

We investigated the drug loading and release capacities of the PBCA MB synthesized with the different surfactants. Coumarin 6 was loaded into the MB shells after synthesis according to a previously established protocol that exploits hydrophobic interactions between the drug and the polymer chains, and washing steps were implemented to remove unencapsulated molecules from the drug-loaded MB (Fig. 5a).^64^ Coumarin 6 was selected as a model drug because of its strong fluorescence and high hydrophobicity, which is comparable to that of clinically used anticancer drugs.^67^ Coumarin derivatives also have established clinical relevance, including vitamin K antagonists such as phenprocoumon,^68^ while coumarin 6 is widely employed as a fluorescent model compound in preclinical studies.^69–71^ Accordingly, the present loading approach is primarily suited to hydrophobic compounds with sufficient affinity for the PBCA shell.^72^

As shown in Fig. 5b, O-20 MB and SA-9 MB showed the highest drug loading, with capacities 6.2- and 4.8-fold greater than those of conventional X-100 MB. Although both formulations showed increased shell thickness distributions in Fig. 4b and c, their enhanced loading performance is likely driven by distinct structural features. SA-9 MB remained predominantly spherical, yet displayed the highest surfactant fraction entrapped within the MB shell (Fig. 2f), suggesting a more porous shell structure that may provide additional cavities for drug loading.^25^ In addition, its broad distribution of longer and more polydisperse PBCA chains (Fig. 2e) may further contribute to the increased drug loading capacity.^28^ In contrast, O-20 MB showed a relatively low surfactant fraction and a narrow distribution of shorter PBCA chains. However, this sample also exhibited the highest fraction of particles with asymmetric morphologies (Fig. 4a), which may increase the available surface area^23^ and thereby enhance drug loading. Although the other samples did not show a statistically significant increase in loading, O-10 MB, 15-S-15 MB, and 15-S-20 MB exhibited 1.9-, 2.0-, and 2.4-fold higher loading than the X-100 MB counterparts, respectively. However, upon exposure of PBCA MB to US pulses, drug release from the burst MB shells was similar for all samples, reaching ∼60% (Fig. 5c), consistent with our previous reports.^22,66^

**Fig. 6:**
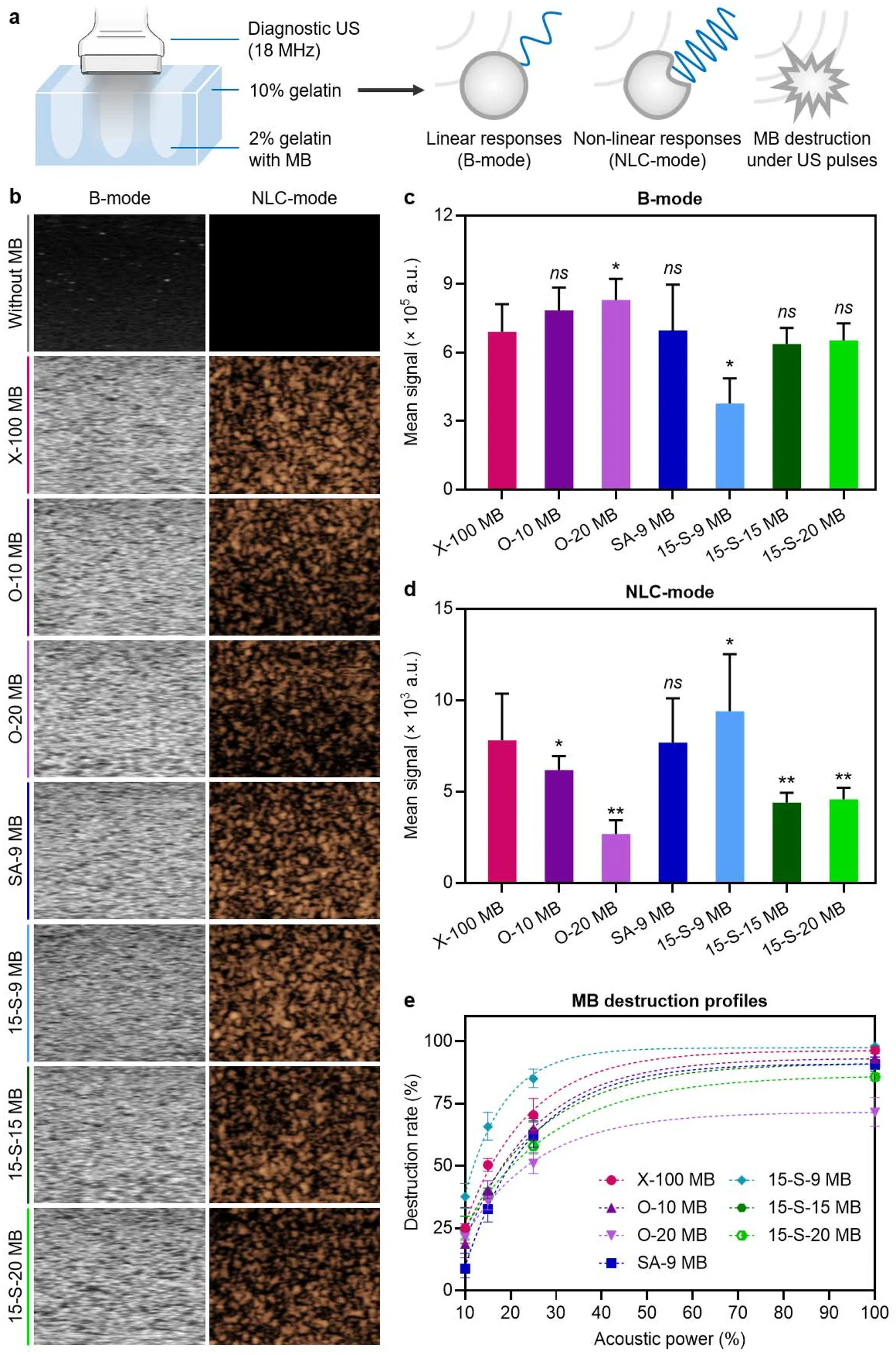
Acoustic characterization of surfactant-based PBCA MB. (a) Schematic representation of the study setup and experiment readouts. (b) Representative B-mode and non-linear contrast mode (NLC-mode) images of PBCA MB at a low acoustic power of 4%. (c and d) Mean signal intensities for MB in the (c) B-mode mode, and (d) NLC-mode at a 4% acoustic power. (e) MB destruction profiles under US pulses of increasing acoustic power fitted with a one-phase decay model of the experimental data. Values represent mean ± standard deviation of three different batches of PBCA MB, each measured in triplicates. (*) and (**) indicate groups that are significantly different with *p* < 0.05 and *p* < 0.01, respectively; (ns) indicates groups that are not significantly different with *p* > 0.05 (one-way ANOVA with *post hoc* Tukey HSD test). Mean MB destruction rates at each presented acoustic power are shown in Fig. S9.

To investigate the influence of greener surfactants on MB acoustic properties, we initially evaluated US responses of the same MB count embedded in gelatin phantoms using a preclinical imaging system with a central frequency of 18 MHz, which is frequently used in small-animal imaging studies (Fig. 6a).^64^ The gelatin phantoms provided a low-cost, stable, tissue-like, and homogeneous medium that minimized motion artifacts.^73,74^ In addition, MB concentration was selected to disperse individual MB in the medium, ensuring that the measured acoustic responses primarily reflected the intrinsic MB features rather than effects from their movement or aggregation.^44^ At low acoustic power of 4%, corresponding to a mechanical index of 0.03, B-mode reflects the baseline echogenicity of MB through predominantly linear, fundamental backscatter, while non-linear contrast (NLC) mode detects amplitude-dependent non-linear scattering and therefore indicates how readily MB depart from linear oscillation, generating unique signals that can be separated from those generated by tissues during *in vivo* examination.^44^ As acoustic power increases, the rate of signal loss reflects MB stability, with faster loss indicating greater susceptibility to acoustic destruction.^75^

All MB samples exhibited strong signals in both modes, while no signal was observed in the absence of MB (Fig. 6b). At low acoustic power, most MB samples showed comparable mean B-mode signal intensities to the X-100 MB used as a control (Fig. 6c). 15-S-9 MB exhibited reduced signal intensity, consistent with their slightly smaller diameter distribution compared to other samples (Fig. 2c and Table S3). In contrast, O-20 MB showed a slight increase in B-mode signals, consistent with their larger diameter distribution. Although MB signals arise from a combination of parameters, including diameter, shell thickness, and viscoelastic properties, we hypothesize that variations in diameter are the primary determinant of B-mode response under the present conditions.^76^

In NLC-mode, differences between formulations were more pronounced, reflecting variations in shell viscoelasticity and non-linear oscillation behavior (Fig. 6d). MB with thicker shells and a lower fraction of smooth, well-formed spheres (*e.g.* O-20 MB and 15-S-20 MB) exhibited weaker NLC responses, consistent with increased damping and reduced non-linear oscillation. By contrast, 15-S-9 MB showed relatively weak B-mode signal but a stronger NLC response, indicating limited fundamental backscatter yet a greater tendency to undergo non-linear oscillation under acoustic excitation. Notably, X-100 MB and SA-9 MB showed comparable responses despite differences in polymer chain length distribution, suggesting competing effects on shell viscoelastic properties.

Under a gradual increase in acoustic power, MB destruction rates were also evaluated (Fig. 6e, mean rates at each acoustic power are presented in Fig. S9). At 10% acoustic power, 15-S-9 MB already showed a higher destruction rate than the other samples, suggesting a lower acoustic threshold for unstable oscillation and subsequent shell disruption. With increasing acoustic power, this formulation consistently retained the highest destruction rates, further indicating its reduced acoustic stability compared with the other samples. The remaining formulations exhibited comparable or greater resistance to increasing acoustic power. Among them, O-20 MB showed the lowest destruction rates at both 25% and 100% acoustic power, consistent with enhanced acoustic stability. This behavior is in line with its comparatively strong B-mode signal and weak NLC response, suggesting efficient fundamental scattering but limited amplitude-dependent non-linear oscillation relative to the other formulations.

Overall, these results suggest that each greener surfactant alternative should be evaluated individually with respect to resulting PBCA MB acoustic performance. Although most formulations showed broadly comparable behavior, some exhibited distinct acoustic signatures, indicating potential application-specific advantages. For example, formulations with strong non-linear response, and relatively high susceptibility to acoustic disruption may be advantageous in US-triggered and image-guided drug delivery, where small molecules can be effectively released from the strongly vibrating MB while being detected in US sonograms,^64^ or contrast-specific imaging workflows that benefit from MB signal isolation, *e.g.* US localization microscopy.^77^ By contrast, more acoustically stable formulations may be better suited to applications requiring sustained signal persistence or tightly controlled cavitation without bursting, such as some brain-focused US protocols or US-guided therapeutic scenarios.^75,78^

In addition, we assessed whether the use of greener surfactants preserved the *in vitro* biocompatibility of PBCA MB using two cell lines commonly employed in toxicological assays (Fig. S10). Human embryonic kidney (HEK293) cells and murine hepatocellular carcinoma (HEPA) 1–6 cells were obtained from the DSMZ-German Collection of Microorganisms and Cell Cultures (Germany). Cytotoxicity was evaluated using lyophilized MB fragments, consisting of PBCA polymeric chains and residual surfactant fractions, to avoid interference from MB buoyancy. Overall, both cell lines tolerated exposure to the different MB fragments well, and all samples showed similar cytotoxicity profiles. These results support the biocompatibility of the surfactant-based PBCA MB platform.

### 2.5 Considerations for microbubble engineering with surfactants

**Fig. 7:**
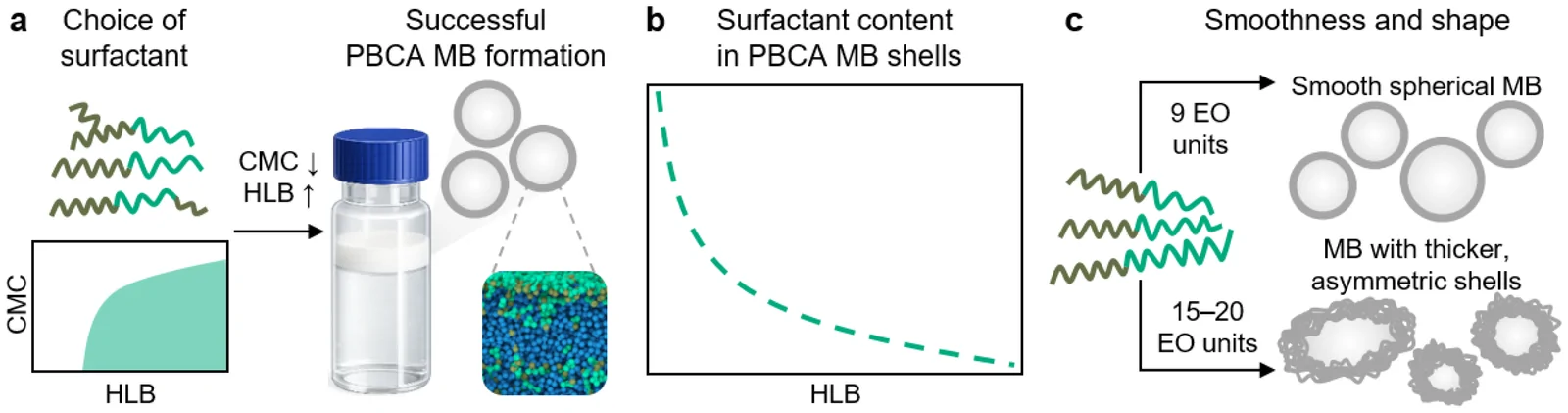
Considerations for PBCA MB engineering with surfactants. (a) A balance between moderate-to-high HLB and relatively low CMC is required to promote successful MB formation, enabling amphiphilic surfactant molecules to effectively stabilize the initial MB templates, followed by polymer chain growth and stabilization of the PBCA MB shells. (b) The surfactant content in PBCA MB shells generally decreases with increasing HLB. (c) The PEG chain length of a surfactant, expressed as the number of EO units, can also influence whether smooth spherical MB or asymmetric morphologies are formed, both of which can be relevant for different applications.

Overall, surfactants should be considered as active structure-directing components in PBCA MB synthesis rather than as passive stabilizers. In the present study, successful MB formation clustered within a physicochemical window defined by sufficiently high HLB and relatively low CMC as schematically illustrated in Fig. 7a, while the structural analysis further suggested that PEG-based surfactants generally required at least about 9 EO units together with a hydrophobic moiety strong enough to counterbalance the hydrophilicity of the chain. Taken together, these observations support the idea that PBCA MB formation depends on a narrow amphiphilic balance: the surfactant must be hydrophilic enough to remain soluble and stabilize the aqueous environment, yet sufficiently surface-active and hydrophobically anchored to adsorb efficiently at the initial gas–liquid interface and support subsequent polymer shell formation. Within that formation window, MB yield appears to be influenced by the architecture of the hydrophobic moiety, with less compact and more flexible hydrophobic tails potentially stabilizing the gas–liquid interface more efficiently than bulkier, more rigid hydrophobic tails, thereby favoring higher MB concentrations.

Surfactant composition can also affect the dispersity of the polymer chains forming PBCA MB shells, with the triblock architecture of Ecosurf SA-9 producing longer and more polydisperse chains than the diblock amphiphiles considered in this work. As highlighted in Fig. 7b, the parent surfactant not only governs templating during synthesis but also becomes part of the final shell: NMR analysis indicated that surfactant incorporation into the shell decreased with increasing HLB, while DPD simulations further suggested preferential enrichment of surfactant at the polymer–water interface. This implies that HLB is relevant not only to synthesis success but also to the internal organization of the final shell. Lower-HLB surfactants may require or retain larger shell fractions, whereas higher-HLB surfactants may accumulate more strongly at the interface than in the shell interior. For sufficiently hydrophobic surfactants, stronger association with the growing PBCA phase could alter surfactant partitioning during polymerization and potentially compromise stabilization of the evolving MB template; however, the present experiments do not resolve this redistribution kinetically. These differences in surfactant partitioning are also likely to influence shell porosity and local free volume, thereby contributing to variations in interfacial composition.

Surprisingly, the hydrophilic segment, particularly PEG chain length expressed as EO number, strongly influenced MB morphology (Fig. 7c). Surfactants with around 9–10 EO units tended to produce predominantly spherical MB, whereas extension of the EO chain to 20 units shifted the population toward more asymmetric morphologies and broader shell-thickness distributions. A plausible explanation is that longer EO chains increase the effective headgroup size and steric footprint of the surfactant, thereby generating a more hydrated and less compact interfacial layer during template polymerization. From an engineering perspective, this suggests that surfactant selection can be separated into at least two design questions: first, whether a given amphiphile can support MB formation at all, and second, whether it biases the resulting shells toward spherical or non-spherical particles.

These interfacial and structural effects translated into distinct performance profiles in the present work and, with further refinement, may support different application scenarios by leveraging the mechanistic effects described here. For instance, the emerging concept of monoacoustic MB for US imaging hypothesizes generating MB populations that are not only narrowly size-distributed but also highly uniform in shell structure and morphology, as deviations from spherical geometry may dampen the acoustic response, while shell irregularities may create local weak points that promote MB destruction.^79–81^ From this viewpoint, MB produced with Triton X-100 appear particularly advantageous, as the combination of a rigid, compact octylphenyl hydrophobic moiety and a hydrophilic segment containing 9 EO units promoted the highest fraction of smooth spheres among the tested samples, hence, yielded a balanced acoustic profile in terms of signal generation and controlled destruction. In parallel, the rapidly growing field of acoustic microrobotics seeks to harness acoustic energy for active actuation and task execution within living systems.^82^ In this context, bubble-based designs that exhibit asymmetry can enable propulsion in controlled settings,^83–85^ rendering asymmetric MB potentially advantageous. Further advances in controlling asymmetric MB shape formation may support the development of acoustic microrobots. MB-based drug delivery applications require drug loading optimization, since the gaseous core occupies most of the MB volume, together with efficient release upon exposure to US pulses.^20^ SA-9 MB and O-20 MB achieved the highest drug loading, but apparently for different reasons: SA-9 MB combined predominantly spherical morphology with the highest surfactant fraction in the shell and a broader distribution of longer PBCA chains, O-20 MB combined low surfactant fraction with shorter chains but a higher proportion of non-spherical particles, which may increase accessible surface area. Although both showed similar drug release profiles in our model experiments, their distinct MB destruction profiles under diagnostic US suggest that these two formulations may be better suited to different application scenarios. Therefore, surfactant engineering should not be framed as the search for one universally optimal amphiphile, but rather as the selection of surfactant architectures that match a desired functional endpoint.

Although this study primarily focused on greener surfactants recently developed for industrial use, the incorporation of surfactants with relevance to biomedical and cosmetic formulations may further support the translational of polymeric MB. In this context, Brij O-10 and the closely related surfactant Brij 97 have been explored in ocular microemulsions and contact-lens hydrogels,^86,87^ transdermal emulsions and lyotropic liquid-crystal systems,^88,89^ and cosmetic formulations^90^ to improve hydrophobic drug loading, sustained and controlled delivery, skin permeation, and formulation stability. Although Brij O-10 has not achieved broad adoption as an established pharmaceutical excipient, its increasing use in experimental biomedical and cosmetic formulations highlights its translational potential. Tergitol 15-S surfactants, particularly Tergitol 15-S-9, have been introduced into biomedical-adjacent applications, including diagnostic sample preparation and viral inactivation,^36,91^ while their relevance in skincare applications remains largely product profile adjacent.^92^ In contrast, Tween surfactants considered in our previous study,^28^ particularly Tween 20, are well-established regulated excipients in approved biomedical products and clinical formulations,^93^ and are increasingly considered as milder alternatives to Triton X-100. Collectively, these observations highlight a key limitation: several greener surfactants were developed for industrial rather than biomedical use, so their suitability for clinical translation requires case-by-case evaluation. Future surfactant design should therefore consider not only the design principles described in this work, but also translational and regulatory perspectives.

## 3 Conclusions

In this study, we used 14 greener non-ionic surfactants to clarify how surfactant chemistry governs PBCA MB synthesis and performance. Six surfactants enabled successful MB formation, allowing us to identify key mechanistic relationships between surfactant structure and MB properties. The architecture of the hydrophobic moiety primarily influenced MB yield, whereas increasing PEG chain length promoted less spherical morphologies and broader shell-thickness distributions. In addition, surfactant incorporation into the MB shell depended on the hydrophilic–lipophilic balance of the parent surfactant, and dissipative particle dynamics simulations indicated preferential surfactant enrichment at the polymer–water interface. Together, these findings support the concept that surfactants play an active templating role during PBCA MB formation, rather than serving only as passive stabilizers. Distinct surfactant architectures also translated into distinct functional outcomes, including enhanced drug loading and tunable acoustic behavior, indicating that surfactant selection can be used to tailor MB morphology, shell composition, drug loading capacity, and acoustic response for different applications. Overall, this work provides mechanistic insight into the role of surfactants in PBCA MB synthesis and establishes greener surfactant engineering as a practical framework for application-oriented MB design.

## Author contributions

R. A. B. and R. M. P. designed the research. R. A. B. and S. A. S. carried out the synthesis and characterization of the polymeric microbubbles. R. A. G. performed the theoretical simulations. Y. L. and M. M. assisted with the experiments. J. K. and M. P. performed the SEC and NMR analyses, respectively, and C. B. and S. R. carried out the microscopy. L. D. L., K. L., I. I. P., F. K., and T. L. provided scientific guidance. R. A. B. wrote the original draft of the manuscript. All authors reviewed the manuscript and approved the final version.

## Conflicts of interest

F. Kiessling and T. Lammers are among the co-founders of the SonoMAC GmbH that produces polymeric microbubbles. F. Kiessling is a consultant of Fujifilm Visualsonics and of BRACCO. F. Kiessling, T. Lammers, R. M. Pallares, R. A. Barmin and M. Moosavifar are named inventors on patents related to polymeric microbubble technology. The other authors have no relevant conflicts of interest to declare.

## Abbreviations

PBCA: Poly(butyl cyanoacrylate)
MB: Microbubbles
US: Ultrasound
MW: Molecular weight
HLB: Hydrophilic–lipophilic balance
CMC: Critical micelle concentration
PEG: Poly(ethylene glycol)
EO: Ethylene oxide
PO: Propylene oxide
BCA: Butyl cyanoacrylate
X-100 MB: Triton X-100 generated microbubbles
O-10 MB: Brij O-10 generated microbubbles
O-20 MB: Brij O-20 generated microbubbles
SA-9 MB: Ecosurf SA-9 generated microbubbles
15-S-9 MB: Tergitol 15-S-9 generated microbubbles
15-S-15 MB: Tergitol 15-S-15 generated microbubbles
15-S-20 MB: Tergitol 15-S-20 generated microbubbles
SEC: Size-exclusion chromatography
NMR: Nuclear magnetic resonance spectroscopy
*M*_n_
: Number-average molar mass
*M*_w_
: Weight-average molar mass
DPD: Dissipative particle dynamics
cryoSEM: Scanning electron cryo-microscopy
CLSM: Confocal laser scanning microscopy
NLC-mode: Non-linear contrast mode

## Supplementary Material

GC-028-D6GC02897G-s001

## Data Availability

The data supporting this article, together with materials and methods, have been included as part of the supplementary information (SI). Supplementary information is available. See DOI: https://doi.org/10.1039/d6gc02897g.
