# Supplementary material for: Green surfactants reveal mechanistic principles governing polymeric microbubble formation and function
Source: Green Chem. 2026 Aug 28;28(35):14515–31. doi: 10.1039/d6gc02897g (PMC13523131; doi:10.1039/d6gc02897g)
Supplement: Supplementary file 1 [file GC-028-D6GC02897G-s001.pdf]

Supplementary Information (SI) for Green Chemistry.
This journal is © The Royal Society of Chemistry 2026

– SUPPORTING INFORMATION –

Green Surfactants Reveal Mechanistic Principles Governing Polymeric Microbubble Formation and Function

Roman A. Barmin,[1,⁋] Sabina A. Saraolu,[1,2,3,⁋] Rustam A. Gumerov,[4] Yanchen Li,[1] MirJavad Moosavifar,[1] Jens Köhler,[4] Michael Pohl,[4] Céline Bastard,[4,5,6] Stephan Rütten,[7] Laura De Laporte, [4,5,6] Katharina Landfester,[8] Igor I. Potemkin,[4] Fabian Kiessling,[1,*] Twan Lammers,[1,*] and Roger M. Pallares[1,*]

[1] Institute for Experimental Molecular Imaging, RWTH Aachen University Hospital, Aachen 52074, Germany

[2] Department of Bioengineering and Biotechnology, Faculty of Medical Engineering, National University of Science and Technology POLITEHNICA Bucharest, 060042 Bucharest, Romania

3 Center for Molecular and Cellular Bioengineering, TUD Dresden University of Technology, Dresden 01307, Germany

[4] DWI – Leibniz Institute for Interactive Materials, Aachen 52074, Germany

[5] Institute of Technical and Macromolecular Chemistry, RWTH Aachen University, Aachen 52074, Germany

[6] Department of Advanced Materials for Biomedicine, Institute of Applied Medical Engineering, RWTH Aachen University, Aachen 52074, Germany

[7] Electron Microscope Facility, RWTH Aachen University Hospital, Aachen 52074, Germany

[8] Max Planck Institute for Polymer Research, Mainz 55128, Germany

⁋ These authors contributed equally to this work.

*Corresponding authors rmoltopallar@ukaachen.de; tlammers@ukaachen.de; fkiessling@ukaachen.de

Table of Contents

EXPERIMENTAL SECTION S3
Materials S3
PBCA MB synthesis S3
Quantification of concentrations and diameter distributions of PBCA MB S3
Coumarin 6 drug loading in PBCA MB S4
Characterization of polymer chains and entrapped surfactant amounts within PBCA MB shells S4
Dissipative particle dynamics simulations S5
Characterization of PBCA MB morphology, shell thickness, and sphericity S8
Quantification of drug loading and drug release S8
In vitro acoustic characterization of PBCA MB S9
In vitro cytotoxicity assay S10
Statistical analysis S10
Table S1. Surfactant concentrations and volumes used for PBCA MB synthesis and storage S11
Table S2. Surfactant solubility and its influence on MB synthesis and retrieval S12
Table S3. Mean diameters and concentrations of PBCA MB S13
Table S4. Hansen solubility parameters (HSP) used in the DPD simulations S14
Table S5. Structural formulas of the coarse-grained models of different surfactant types S15
Table S6. DPD interaction parameters used in the simulations. S16
Figure S1. NMR spectra of Triton X-100 and X-100 MB S17
Figure S2. NMR spectra of Brij O10 and O-10 MB. S18
Figure S3. NMR spectra of Brij O20 and O-20 MB S19
Figure S4. NMR spectra of Ecosurf SA-9 and SA-9 MB S20
Figure S5. NMR spectra of Tergitol 15-S-9 and 15-S-9 MB S21
Figure S6. NMR spectra of Tergitol 15-S-15 and 15-S-15 MB S22
Figure S7. NMR spectra of Tergitol 15-S-20 and 15-S-20 MB S23
Figure S8. Correlation between surfactant EO number and the smooth sphere fraction S24
Table S7. Drug molecules per individual MB and drug loading per 1×10^9 MB S25
Figure S9. Destruction rates of the PBCA MB. S26
Figure S10. *In vitro* assessment of the PBCA chains S27
REFERENCES S28

EXPERIMENTAL SECTION

Materials

Butyl cyanoacrylate (BCA) was provided by Chemence (UK). Triton X-100, Brij O10, Brij O20, Ecosurf SA-4, Ecosurf SA-7, Ecosurf SA-9, Ecosurf EH-9, Tergitol TMN 6, Tergitol TMN 10, Tergitol 15-S-9, Tergitol 15-S-15, Tergitol 15-S-20, Triton CG-110, mannide monooleate, sucrose stearate, gelatin, coumarin 6, and dimethyl sulfoxide (DMSO) were purchased from Sigma-Aldrich (Germany). Dulbecco's modified Eagle's medium (DMEM), phosphate-buffered saline (PBS), fetal bovine serum (FBS), penicillin-streptomycin (P/S) antibiotics, ethylenediaminetetraacetic acid (EDTA), trypsin and poly-D-Lysine were purchased from Thermo Fisher Scientific (Germany). Deionized (DI) water was produced by a PURELAB flex 2 device from ELGA LabWater (Celle, Germany) and used for all experiments. All other reagents were of analytical grade.

PBCA MB synthesis

PBCA MB were synthesized according to protocols adapted from our previous studies.[1,2] Surfactant solutions were prepared by dissolving the selected detergent in 300 mL DI water to a final concentration of 16.7 mM, which is equivalent to the 1% (w/v) used for Triton X-100, and adjusting the pH to 2.5 by dropwise addition of 1 M HCl. Table S1 lists the surfactant volumes corresponding to the desired surfactant concentration, and Table S2 summarizes the surfactant solubility under the specified conditions. Next, 3 mL of BCA monomer was added dropwise at room temperature, and the mixture was stirred for 1 hour at 10,000 RPM using an Ultra-Turrax T50 (IKA-Werke, Germany) with the tip positioned at the gas-liquid interface. The resulting suspension, containing polydisperse MB, was purified by three successive centrifugation cycles (500 rpm, 20 min each), during which MB floated to the air–liquid interface and the underlying liquid was aspirated. After each cycle, the floated MB cake was redispersed in 40 mL of storage solution in 50 mL Falcon tubes. The storage solution was adjusted to pH 7.0 by dropwise addition of 1 M NaOH to yield a final surfactant concentration of 0.3 mM, as listed in Table S1. Finally, the MB suspensions were combined in a single 100 mL glass bottle prefilled with 50 mL of storage solution and gently mixed on a roller mixer to ensure homogeneous dispersion.

Quantification of concentrations and diameter distributions of PBCA MB

The diameter distribution and concentration of PBCA MB were analyzed using a Multisizer 4 (Beckmann Coulter, Germany) with a 30 µm sensor aperture covering a size range of 0.6 to 18

μm. 5 μL of each MB sample was diluted in 20 mL of ISOTON II® isotonic solution (Beckmann Coulter, Germany) and measured in volumetric mode with analytical volume of 50 μL. For each sample, 20 mL of isotonic solution was measured with the same procedure and subtracted from the measurement result as background noise. All measurements were performed at room temperature, and the mean diameter and concentration values are listed in Table S3.

Coumarin 6 drug loading in PBCA MB

Coumarin 6 was loaded into PBCA MB as a model drug following our previously established method.[1,2] Briefly, 5 mg of coumarin 6 was dissolved in 100 μL of DMSO and added to a 10 mL of MB suspension with a concentration of 10^9 MB/mL in storage solution. The mixtures were then stirred at 40 RPM for 24 h. Next, the samples were left undisturbed overnight to let MB float while being covered with aluminium foil to avoid light exposure. The aqueous solutions underneath the MB cakes were replaced with fresh storage solution multiple times to remove the unloaded drug until no free drug was detected in the solution via fluorescence spectrometry with a TECAN Infinite Pro M200 (Tecan, Switzerland). Finally, the drug-loaded MB were resuspended in 10 mL of storage solution and stored in the dark for further characterization. All procedures were performed at room temperature and under light-protected conditions to prevent photobleaching.

Characterization of polymer chains and entrapped surfactant amounts within PBCA MB shells

SEC (size-exclusion chromatography) and proton NMR (nuclear magnetic resonance spectroscopy) were performed to analyze the shell composition. Prior to measurements, MB suspensions were washed with DI water to minimize the presence of freely dispersed surfactants. The MB suspensions were subsequently lyophilized using a benchtop freeze dryer (CHRIST Alpha 2-4 LD plus, Martin Christ Freeze-Drying Systems GmbH, Germany) to obtain dry polymer powders corresponding to the MB shells.

Molecular weights ($M_{n,SEC}$ and $M_{w,SEC}$) and molecular weight distributions (M_w/M_n) were determined by size-exclusion chromatography (SEC). SEC analyses were carried out using chloroform ($CHCl_3$) (≥ 99.8%, stabilized with 2-methyl-2-butene, HiPerSolv Chromanorm® HPLC grade, VWR) as the eluent. The machine was equipped with an HPLC-pump (1260 Infinity II, Agilent Technologies), a refractive index detector (RID, 1260 Infinity II, Agilent Technologies), an UV-Detector (VWD, 1260 Infinity II, Agilent Technologies) and an evaporative light scattering detector (Sedex 85, Sedere). The samples contained 250 mg/L 3,5-di-*tert*-4-butylhydroxytoluene (BHT, ≥ 99%, Fluka) as the internal standard. One pre-column

(8 × 50 mm) and four SDplus gel columns (8 × 300 mm, MZ Analysentechnik) were applied at a flow rate of 1.0 mL/min at 40 °C. The diameter of the gel particles measured 5 µm, the nominal pore widths were 50, 10^2, 10^3 and 10^4 Å. Calibration was performed using narrowly distributed poly(styrene) standards (Agilent Technologies). Results were evaluated using the Agilent Technologies WinGPC software (Version 1.0).

NMR spectra of the shell materials and corresponding surfactants were recorded on a Bruker Avance III 400 MHz spectrometer (Bruker, Germany) using deuterated chloroform as the solvent. The relative amount of surfactant incorporated into the PBCA MB shells was quantified by comparing integral values of selected ^{1}H NMR signals of PBCA and surfactant.

Dissipative particle dynamics simulations

We employed dissipative particle dynamics (DPD) simulation method in its standard form.[3–5] For DPD, solvent molecules, as well as the polymer segments, are explicitly included and represented in terms of spherical beads, whereas each bead usually comprises a group of atoms. The beads of equal mass and size interact with each other by a pairwise additive force:

$$F_i = \sum_{j \neq i} (F_{ij}^C + F_{ij}^D + F_{ij}^R + F_{ij}^B) \quad (1)$$

where F_{ij}^C is a conservative force responsible for repulsion via soft potential characterized by the parameter a_{ij}:[3] the larger the value of a_{ij}, the stronger the repulsion between the ith and the jth beads. F_{ij}^D and F_{ij}^R are the dissipative and random forces, respectively, which serve as heat sink and source and are specified by a friction coefficient λ and the noise amplitude σ. F_{ij}^B is a bond force that keeps the polymer beads together and that is specified by the spring constant k_s and the equilibrium bond length r_0. The sum runs among all the beads in the system. The first three forces on the right-hand-side of equation 1 act only within a certain cutoff radius r_c, which usually serves as the characteristic length scale unit.[5] The non-bonded forces in equation 1 are given by the following expressions:

$$\mathbf{F}_{ij}^{C} = \begin{cases} a_{ij}(1-r_{ij})\bar{\mathbf{r}}_{ij}, & r_{ij} < 1 \\ 0, & r_{ij} \geq 1 \end{cases} \quad (2)$$

$$\mathbf{F}_{ij}^{D} = -\lambda[\omega(r_{ij})]^2(\mathbf{v}_{ij} \cdot \bar{\mathbf{r}}_{ij})\bar{\mathbf{r}}_{ij} \quad (3)$$

$$\mathbf{F}_{ij}^{R} = \sigma\omega(r_{ij})\xi_{ij}\Delta t^{-1/2}\bar{\mathbf{r}}_{ij} \quad (4)$$

Here $\bar{\mathbf{r}}_{ij} = (\mathrm{r}_i - \mathrm{r}_j)/r_{ij}$ is the unit vector pointing from the jth to the ith bead, $\omega(r_{ij}) = (1 - r_{ij})$ is a weight function which turns to zero when $r_{ij} \geq 1$, $\mathbf{v}_{ij} = \mathbf{v}_i - \mathbf{v}_j$ is the relative velocity of the beads

i and j, ξ_{ij} is a zero-mean normally distributed random variable and Δt is a simulation timestep. The bond F_{ij}^{B} force is described by the harmonic potential:

$$E^{B} = \tfrac{1}{2} k_{s} (r - r_{0})^{2} \quad (5)$$

The momentum for each pair of beads is preserved. To satisfy the fluctuation-dissipation theorem, a relation $\sigma^2 = 2k_BT\lambda$ must be provided[4] while the value of λ is set to 4.5 for the decent rate of equilibration of the temperature. The evolution of the system is described by N equations of motion expressed through the second Newton's law, $m\, \mathrm{d}v_i / \mathrm{d}t = F_i$. All quantities are measured in units of the mass of the bead, m, thermal energy, k_BT, and the cutoff radius of the interaction potential, r_c. For convenience, the quantities are fixed as $m = k_BT = r_c = 1$, so that the characteristic timescale is defined as $\tau = r_c\, (m/k_BT)^{1/2}$ and also equals 1.[5]

Before the construction of the MB shell model, the simulation parameters must be first related to the physical time and space scales. We start with the determination of the characteristic length r_c, defined as $r_c = (\rho V_{\mathrm{DPD}})^{1/3}$ where ρ is the number density set to 3, and V_{DPD} is the volume of a single DPD bead. For aqueous systems, a convenient approach is to define a water mapping number, N_m so that each water bead corresponds on average to N_m water molecules. Hence, the characteristic mass of this type of beads will be multiple to 18 Da and the molecular volume v_m will be approximately multiple to 30 Å^3.[6] Since the mass of a single BCA unit is 153 Da respectively, a closest analogue of water molecules group by mass should include $N_m = 8$ molecules (m = 144 Da). Thus, this gives V_{DPD} = 240 Å^3 and $r_c \approx 0.9$ nm.

The next step in the coarse-graining procedure lies in the determination of interactions between PBCA chains, surfactants, and water. Since $\rho = 3$, the interaction parameters a_{ij} can be mapped onto the Flory-Huggins parameters using a linear relation[5]

$$a_{ij} \approx a_{ii} + 3.27\chi_{ij} \quad (6)$$

where $a_{ii} = 25$ for any two beads of the same type. In turn, the calculation of Flory-Huggins parameters can be done through Hansen solubility parameter:[7]

$$\chi_{ij} = \frac{\alpha V_{\mathrm{DPD}} \left((\delta_i^d - \delta_j^d)^2 + 0.25(\delta_i^p - \delta_j^p)^2 + 0.25(\delta_i^h - \delta_j^h)^2 \right)}{k_B T} \quad (7)$$

where α is a numerical coefficient usually taken as 0.6 for polymer systems,[8] and δ^d, δ^p, δ^h are the dispersion, polar and hydrogen bonding Hansen solubility parameters (HSP). Along with PBCA (represented as beads of types A), the simulated systems include the water beads of type W, and, mainly, the various types of surfactant molecules modelled as substances with hydrophobic heads (of types H_I, where index I denotes the type of the head), hydrophilic PEG

tails (or middle blocks for certain cases, type T), and PO tails for Ecosurf SA-9 surfactant (type P). Sugar-based surfactants were omitted from the DPD simulations because their behavior is governed by specific hydrogen-bonding and stereochemical effects that may not be captured accurately by the model. For A and most H types, the solubility parameters were calculated using van Krevelen's method.[9] For hydrophilic beads, the HSP were selected as for linear PEG polymers.[7] In turn, the HSP for water and PO tails were also taken from the literature. The values of solubility parameters are summarized in Table S4. In addition, for convenience, the structural formulas of all simulated surfactants are done in Table S5.

Then, according to equations 6 and 7 at T = 298.15 K we obtained the DPD repulsion parameters a_{ij} together with the Flory-Huggins parameters (Table S6). In contrast to our previous publication,[2] a numerical coefficient α in equation 7 was taken as 0.8 for the head-tail interactions. At the same time, the DPD head-tail interaction parameters were calculated via modified version of equation 6 for polymers of blocky sequence. All of these modifications were done in order to provide a stronger repulsion between surfactants' heads and tails.[10] In addition, the surfactant tail-water interactions were set as 26.3 in order to provide the hydrophilicity.[11] Finally, the PO-water interactions were set as 32 in order to provide mild hydrophobicity.

The PBCA MB shell is modeled as a polymer film containing chains of equal length $N = 32$ segments. Along with PBCA chains, the film contains a certain amount of each surfactant. Besides, the values for all bonded interactions were selected as for flexible chains, namely $k_s = 100$ and $r_0 = 0.7$. As we already stated in our previous publication,[2] we also examined the effects of chain rigidity on the morphology of the shell by introducing the angular potential and found no significant differences in comparison to the case of fully flexible macromolecules. The shell of PBCA microbubble is modeled as a polymer film containing chains of equal length $N = 32$ segments.

All the simulations were performed using the open source software LAMMPS,[12] with an integration time step $\Delta t = 0.02\tau$. The macromolecules were placed in a simulation box with imposed periodic boundary conditions in X and Y axes. The dimensions of the box was $L_x \times L_y \times L_z = 50\ r_c \times 50\ r_c \times 100\ r_c$ (45 nm × 45 nm × 90 nm), and the implicit walls were placed in its upper and lower edges. A film containing PBCA chains and surfactants was placed in the lower half of the simulation box while the upper half was filled with water beads. The film thus represents an upper part of the microbubble shell. The concentration of the surfactants inside the simulation box was taken from the NMR results. Then, the systems were equilibrated during

10×10^6 time steps. Subsequently, the density profiles were plotted along the normal to film's surface based on the snapshots taken each 0.5×10^5 steps.

Characterization of PBCA MB morphology, shell thickness, and sphericity

Scanning electron cryo-microscopy (cryoSEM) and confocal laser scanning microscopy (CLSM) micrographs were used to visualize PBCA MB and measure the shell thickness.

CryoSEM micrographs of PBCA MB were obtained using an FE-SEM 4800 (Hitachi, Germany) equipped with an Alto 2500 cryo-transfer system (Gatan GmbH, Germany). Each sample was frozen in liquid nitrogen, sectioned in a cryochamber, and sublimated for up to 10 min to remove residual water. Micrographs were acquired at 1 kV and 2 µA, and MB characteristics were analyzed using ImageJ software (National Institutes of Health, USA).

For CLSM analysis, PBCA MB loaded with coumarin 6 were mounted in Mowiol (Carl Roth, Germany) on high-precision glass slides (Marienfeld, Germany) and examined using an inverted confocal microscope (TCS SP8 X, Leica Microsystems, Germany) equipped with a Plan-Apochromat 100×/1.40 objective. Imaging was performed at an excitation wavelength of 470 nm with an emission detection range of 491–556 nm. MB shell thickness was measured using Leica Application Suite X 3.7.4.

For the sphericity analysis, CLSM images were exported to ImageJ (National Institutes of Health, USA). Individual MB cross-sections were manually outlined, and their projected area and perimeter were determined. The circularity of each MB was calculated using the ImageJ circularity function:

$$C = \frac{4\pi A}{P^2} \quad (8)$$

where A is the projected area and P is the perimeter. Circularity values approaching 1 indicate highly spherical MB, whereas lower values indicate increasing deviations from a spherical shape.

Quantification of drug loading and drug release

To quantify the loading capacities of PBCA MB, each coumarin 6 loaded sample was diluted 100-fold in DMSO and its fluorescence intensity was measured using a TECAN Infinite Pro M200 (Tecan, Switzerland) at an excitation wavelength of 467 nm and an emission wavelength of 514 nm. The average number of drug molecules encapsulated per MB was determined to allow direct comparison across formulations and to maintain consistency with our previous studies,[1,2,13] in which drug loading was reported on a per-MB basis.

To determine the drug release percentage, each sample was diluted 500-fold in storage solution, accounting for the very low solubility of coumarin 6 in aqueous media, and sonicated for 15 min using an Emmi H60 sonication bath (EMAG AG, Germany). The resulting suspension was then centrifuged at 10,000 rpm for 10 min to remove MB fragments, and the concentration of coumarin 6 in the supernatant was quantified using the TECAN Infinite Pro M200.

The average number of drug molecules encapsulated per MB ($N_{drug/MB}$) and the drug release percentage were calculated using the following equations:

$$N_{drug/MB} = \frac{C_d}{M_d} \times \frac{N_A}{C_{MB}} \quad (9)$$

$$Drug\ release\ percentage = \frac{W_r}{W_d} \times 100\% \quad (10)$$

where C_d is the concentration of encapsulated drug (mg/mL), M_d is the molecular weight of coumarin 6 (350.43 g/mol), N_A is the Avogadro's number (6.022×10^{23} mol^{-1}), C_{MB} is the concentration of each sample (MB/mL), W_r is the weight of drug released in solution after sonication, and W_d is the weight of drug loaded in PBCA MB.

Fluorescence quantification was performed after strong sample dilution and, for loading measurements, dissolution in DMSO, minimizing potential interference from residual surfactant.

In vitro **acoustic characterization of PBCA MB**

The acoustic properties of the MB were evaluated using a Vevo 3100 preclinical US imaging system (VisualSonics, Canada) after embedding the MB in gelatin phantoms. Briefly, for each sample, a mixture containing 2% (w/v) gelatin and 1×10^5 MB/mL was prepared to avoid acoustic shadowing. The mixture was then embedded in 10% (w/v) gelatin molds and stored at 4 °C overnight. The resulting phantoms were imaged using an MX250 transducer operating at a center frequency of 18 MHz in both brightness (B-mode) and non-linear contrast (NLC-mode) regimen, where NLC-mode is based on pulse amplitude modulation. Initially, the signal intensity of each sample was measured at 4% power (mechanical index, MI = 0.03). At this setting, the peak negative pressure was estimated to be approximately 100 kPa.[14,15] Subsequently, a second sonogram was recorded at the same 4% power after each sample had been exposed to higher-power ultrasound (10%, 15%, 25%, and 100%, corresponding to mechanical indices of 0.07, 0.105, 0.175, and 0.7, respectively) for 5 s. The MB destruction rate was calculated as follows:

$$MB\ destruction\ rate\ (\%) = \frac{A - B}{A} \times 100\% \quad (11)$$

where A is the mean signal intensity of the sample before higher acoustic power (recorded at 4% power), and B is the mean signal intensity of the sample after higher acoustic power (recorded at 4% power). The mean signal intensities were measured in a region of interest of 25 mm^2 at the probe focus area (set at 10 mm depth) with the Vevo LAB software. Each measurement was performed with six independent replicates. The relationship between increasing acoustic power and MB destruction rate was analyzed by fitting the data to a one-phase decay model.

In vitro cytotoxicity assay

Human embryonic kidney (HEK) 293 cells and murine hepatocellular carcinoma (HEPA) 1-6 cells were obtained from the DSMZ-German Collection of Microorganisms and Cell Cultures (Germany) and used for the in vitro cytotoxicity test of PBCA MB shell material synthesized with different surfactants. HEK 293 cells were cultured in MEM, while HEPA 1-6 cells were cultured in DMEM, both supplemented with 10% FBS and 1% penicillin/streptomycin at 37 °C with 5% CO_2. To avoid MB buoyancy in the presence of cells, PBCA MB were first lyophilized as described above. The resulting polymer powders were dissolved in DMSO to obtain a stock concentration of 6.25 mg/mL. Samples containing various concentrations of PBCA chains (0.5–6.25 µg/mL) were then incubated with cells for 24 h, ensuring that the DMSO content in the culture medium did not exceed 1%. Cell proliferation was subsequently assessed using a standard XTT assay. To prepare the reaction solution for one 96-well plate, 0.1 mL of activation solution was mixed with 5 mL of XTT reagent. Subsequently, 50 µL of the reaction solution was added to each well. The plate was incubated at 37 °C for 4 h. After incubation, optical density values at 450 nm were measured using a microplate reader (BioTek, Winooski, VT, USA). Absorbance values were converted to percentage viability, with the control group (cells incubated with 1% DMSO alone) defined as 100% viability, and all other values calculated relative to this control. To determine cell viability in the XTT assay, the following equation was used:

$$Viability\ (\%) = \frac{Experimental\ value}{Positive\ control\ value} \times 100\%\ (12)$$

Statistical analysis

For each sample, three different batches were synthesized, and each measurement was performed at least three times. Results are reported as mean ± standard deviation. ANOVA analysis with post hoc Tukey HSD test or two-way ANOVA analysis were performed with the

GraphPad Prism 10. p-values lower than 0.05 were considered as statistically significant; (ns), (*), and (**) indicate groups with $p > 0.05$, $p < 0.05$, and $p < 0.01$, respectively.

**Table S1.** Surfactant concentrations and volumes used for PBCA MB synthesis and storage.				
**Surfactant**	**Amount for MB synthesis (mM)**	**Amount for MB synthesis (mL)**	**Amount for MB storage (mM)**	**Amount for MB storage (mL)**
Triton X-100	16.7	3.00	0.3	0.374
Brij O10	16.7	3.52	0.3	0.438
Brij O20	16.7	5.33	0.3	0.665
Ecosurf SA-4	16.7	3.00	0.3	0.374
Ecosurf SA-7	16.7	3.66	0.3	0.457
Ecosurf SA-9	16.7	4.04	0.3	0.503
Ecosurf EH-9	16.7	3.32	0.3	0.413
Tergitol TMN 6	16.7	2.69	0.3	0.329
Tergitol TMN 10	16.7	3.26	0.3	0.406
Tergitol 15-S-9	16.7	2.94	0.3	0.366
Tergitol 15-S-15	16.7	4.24	0.3	0.528
Tergitol 15-S-20	16.7	4.41	0.3	0.549
Triton CG-110	16.7	1.40	0.3	0.172
Mannide monooleate	16.7	2.30	0.3	0.286
Sucrose stearate	16.7	3.02	0.3	0.376

Table S2. Surfactant solubility and its influence on MB synthesis and retrieval.

Surfactant	Dissolution in MB synthesis solution	Dissolution in MB storage solution	MB retrieval after the synthesis
Triton X-100	Possible	Possible	Possible
Brij O10	Possible	Possible	Possible
Brij O20	Possible	Possible	Possible
Ecosurf SA-4	Possible	Possible	Not possible
Ecosurf SA-7	Possible	Possible	Not possible
Ecosurf SA-9	Possible	Possible	Possible
Ecosurf EH-9	Possible	Possible	Not possible, foam formed
Tergitol TMN 6	Possible	Possible	Not possible, foam formed
Tergitol TMN 10	Possible	Possible	Not possible, foam formed
Tergitol 15-S-9	Possible	Possible	Possible
Tergitol 15-S-15	Possible	Possible	Possible
Tergitol 15-S-20	Possible	Possible	Possible
Triton CG-110	Possible	Possible	Not possible, foam formed
Mannide monooleate	Not possible, precipitate formed	Possible	Not possible
Sucrose stearate	Not possible	Possible	Not possible

Table S3. Mean diameters and concentrations of PBCA MB produced with the different surfactants. Values represent mean ± standard deviation of three different batches of PBCA MB, measured in triplicates.

Sample	Concentration (× 10^9 MB/mL)	Mean diameter (µm)
X-100 MB	1.01 ± 0.30	2.6 ± 0.9
O-10 MB	6.30 ± 0.50	2.2 ± 0.6
O-20 MB	5.96 ± 0.43	2.7 ± 1.0
SA-9 MB	1.42 ± 0.17	2.4 ± 0.8
15-S-9 MB	0.40 ± 0.05	1.8 ± 0.4
15-S-15 MB	3.48 ± 0.47	2.3 ± 1.0
15-S-20 MB	4.72 ± 0.60	2.5 ± 1.0

Table S4. Hansen solubility parameters (HSP) used in the DPD simulations.			
**Substance**	δ^d	δ^p	δ^h
PBCA (A)[a]	16.4	7.9	7.9
X-100 – head (H^X)[a]	16.9	0.5	0.0
O-10 and O-20 – head (H^O)[a]	15.9	0	0
SA-9 – head (H^{SA})[a]	15.7	0	0
15-S-9, 15-S-15, 15-S-20 – head (H^{S1})[a, b]	15.1	0	0
15-S-9, 15-S-15, 15-S-20 – head (H^{S2})[a, b]	15.7	3.7	5.20
Hydrophilic tail (T)[c]	17.0	10.0	5.0
PO tail (P)[c]	16.5	9	7
Water (W)[b]	15.5	16.0	42.3

[a] Calculated using van Krevelen's method[9]

[b] at the selected length scale (r_c = 0.9 nm), the Tergitol S head was modelled as a combination of two types, which are connected to the hydrophilic tail from reference[7]

Table S5. Structural formulas of the coarse-grained models of different surfactant types.

Surfactant	Formula	Notes
Triton X-100	$H^{X}T_3$	
Brij O10	$H^{O}{}_{2}T_3$	
Brij O20	$H^{O}{}_{2}T_6$	
Ecosurf SA-9	$H^{SA}T_3P$	The head bead represents a C_9H_{19} radical, which was considered as mean value
Tergitol 15-S-9	$H^{S1}H^{S2}T_3$	The head beads are connected only with the neighboring tail bead. H^{S1} represents C_7H_{15} radical, while H^{S2} represents $C_6H_{14}O$ radical.
Tergitol 15-S-15	$H^{S1}H^{S2}T_4$	
Tergitol 15-S-20	$H^{S1}H^{S2}T_6$	

**Table S6**. DPD interaction parameters used in the simulations.									
a_{ij}	***A***	H^X	H^O	H^{SA}	H^{S1}	H^{S2}	***T***	***P***	***W***
A	25.0	28.3	28.6	28.6	28.6	25.8	25.4	25.1	60.9
H^X		25.0	–[a]	–[a]	–[a]	–[a]	37.8	–[a]	83.3
H^O			25.0	–[a]	–[a]	–[a]	40.0	–[a]	83.5
H^{SA}				25.0	–[a]	–[a]	40.0	30.0	83.5
H^{S1}					25.0	26.6	40	–[a]	83.5
H^{S2}						25.0	30	–[a]	68.7
T							25.0	30	26.3
P								25.0	32
W									25.0

[a] Weren't considered in simulations.

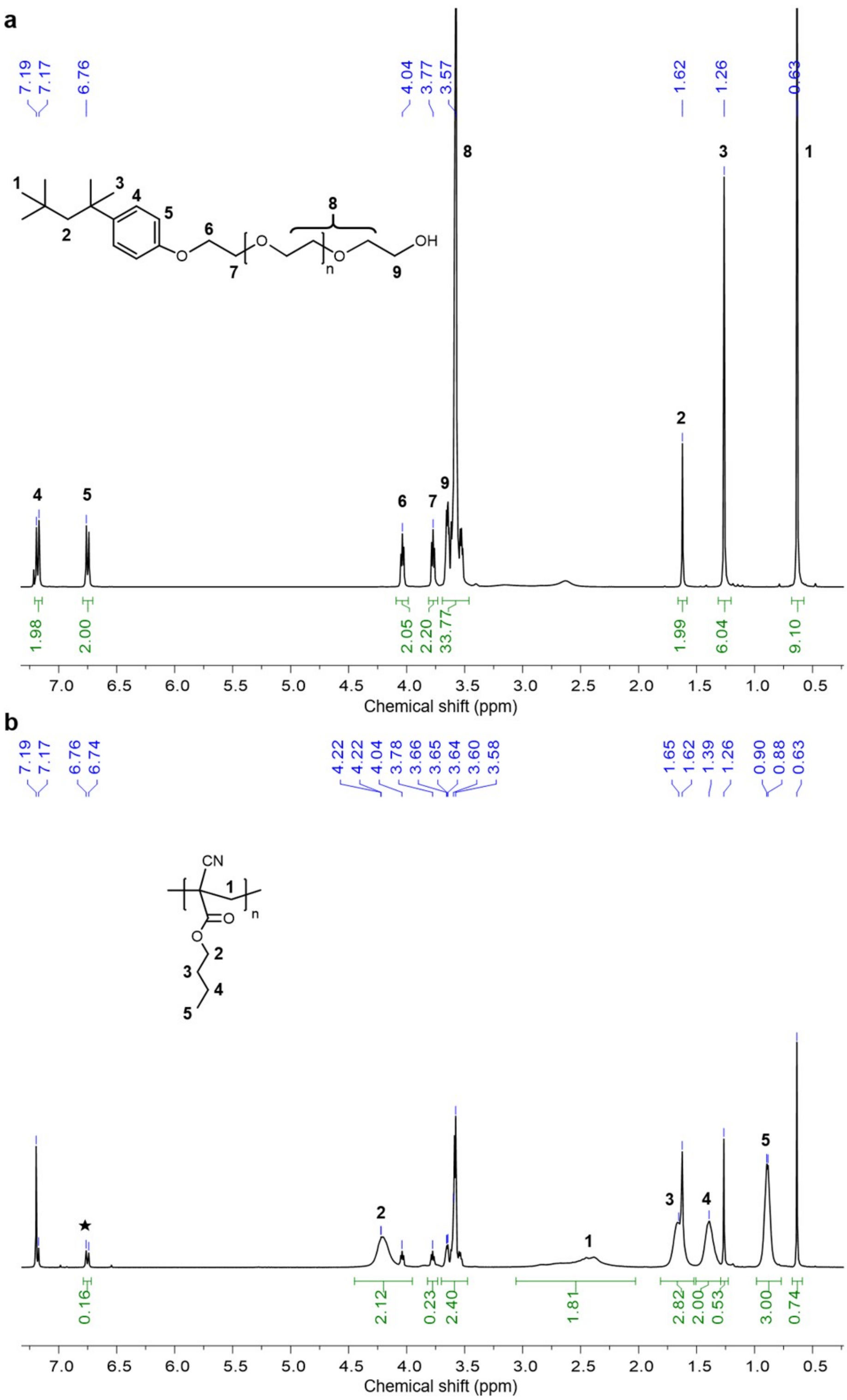

Figure S1. NMR spectra of Triton X-100 and X-100 MB. Spectra of (**a**) Triton X-100 and (**b**) the corresponding PBCA MB sample with peak assignments. The star indicates the signal used to quantify the relative surfactant content in the PBCA MB shell material.

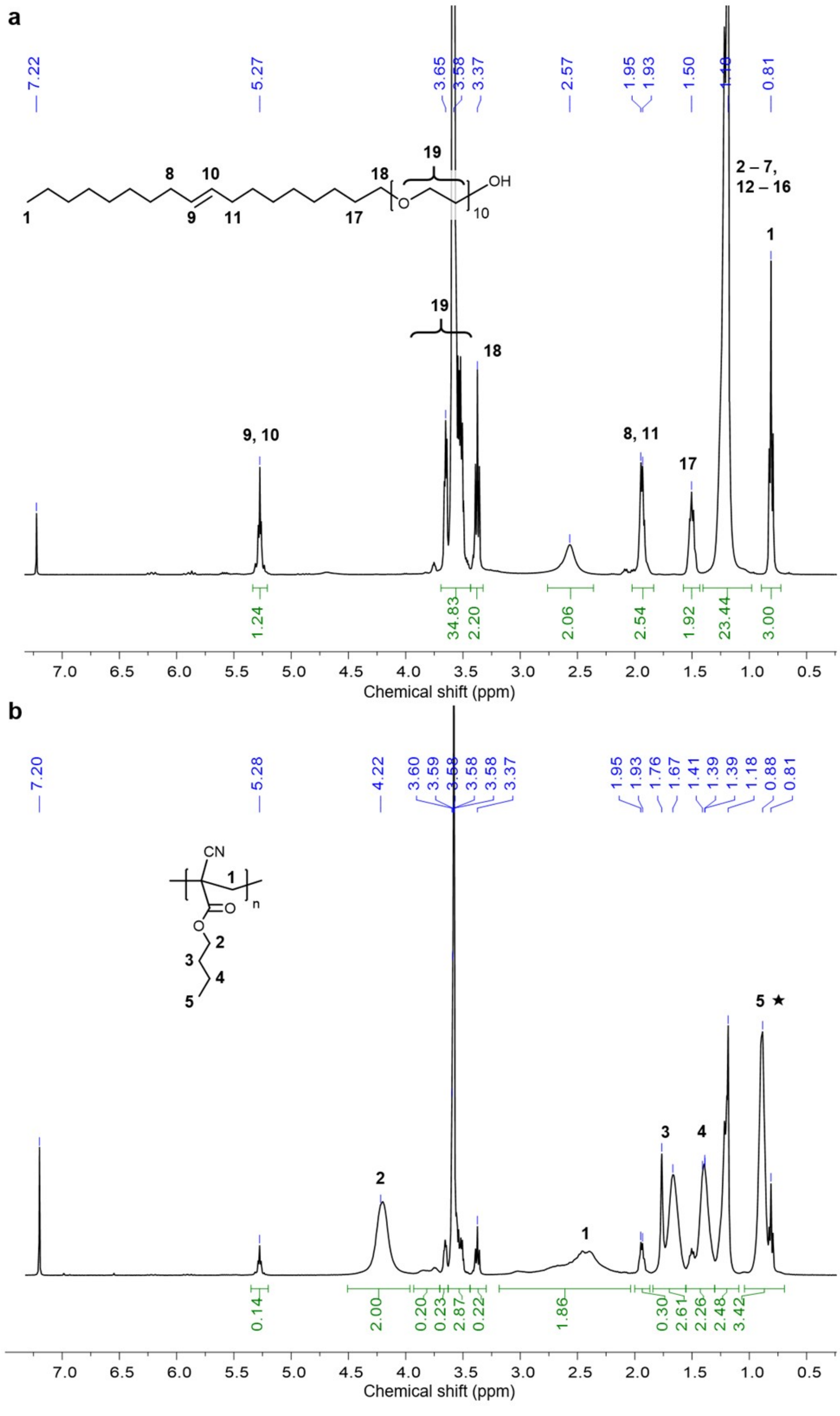

Figure S2. NMR spectra of Brij O10 and O-10 MB. Spectra of (**a**) Brij O10 and (**b**) the corresponding PBCA MB sample with peak assignments. The star indicates the signal used to quantify the relative surfactant content in the PBCA MB shell material.

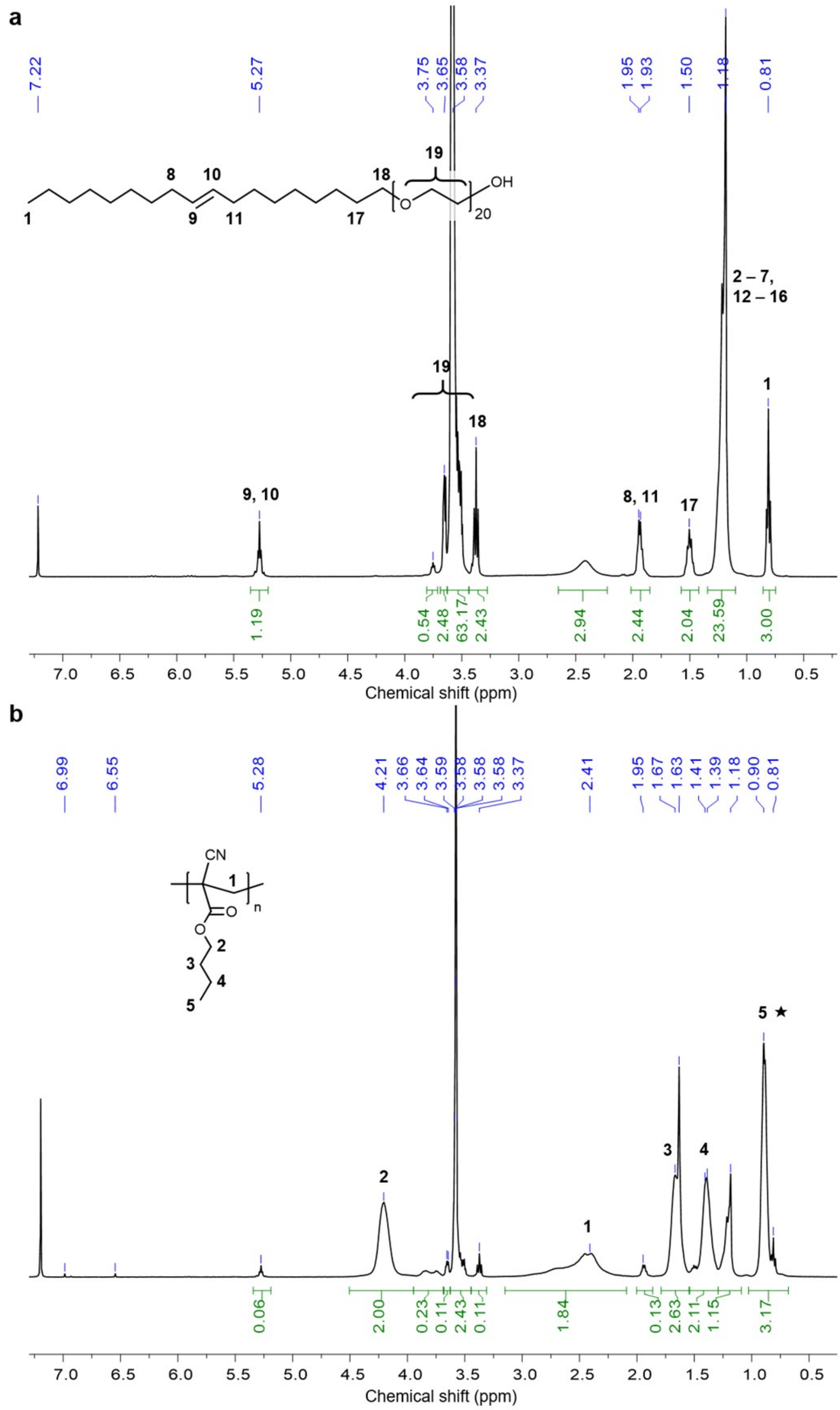

Figure S3. NMR spectra of Brij O20 and O-20 MB. Spectra of (**a**) Brij O20 and (**b**) the corresponding PBCA MB sample with peak assignments. The star indicates the signal used to quantify the relative surfactant content in the PBCA MB shell material.

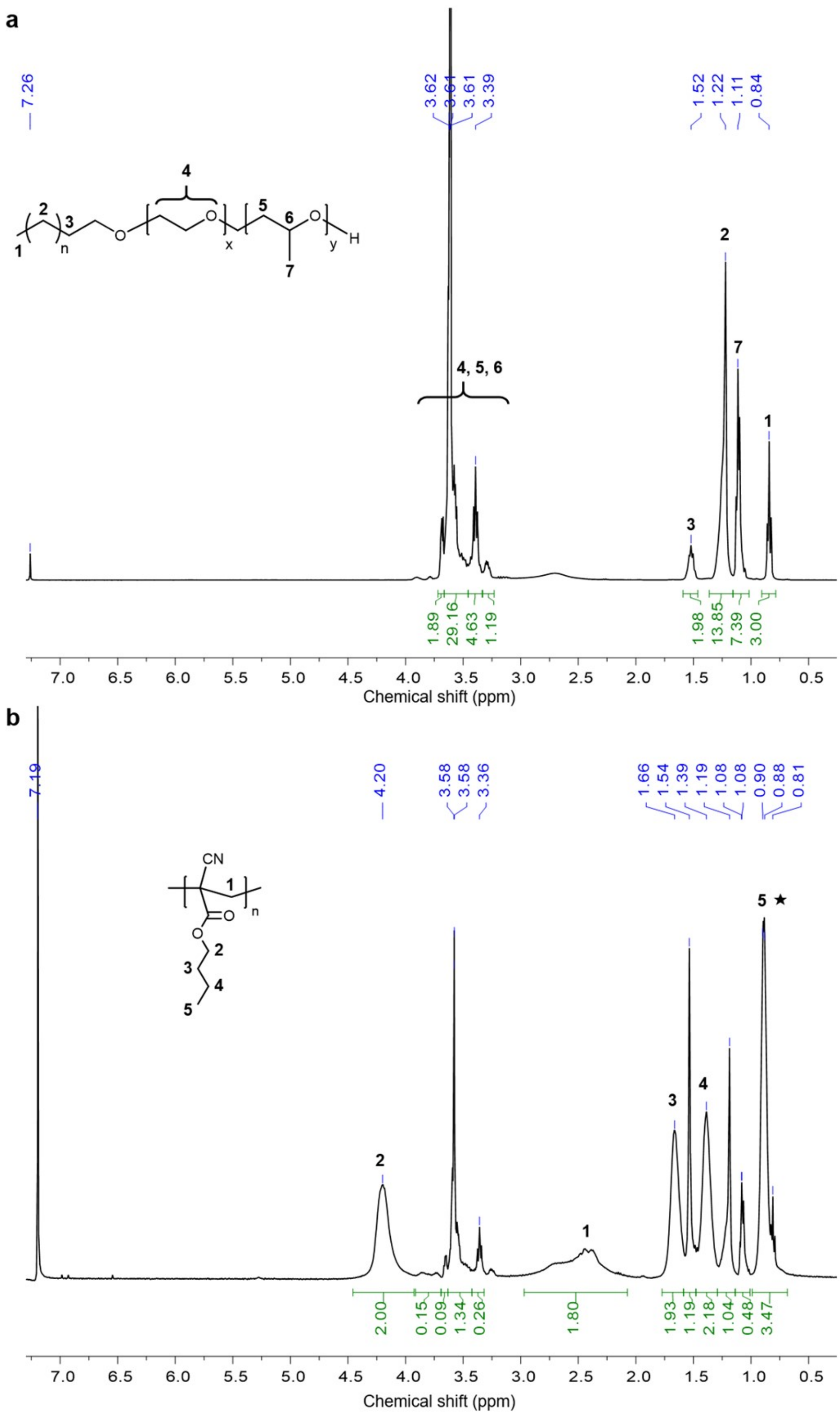

Figure S4. NMR spectra of Ecosurf SA-9 and SA-9 MB. Spectra of (**a**) Ecosurf SA-9 and (**b**) the corresponding PBCA MB sample with peak assignments. The star indicates the signal used to quantify the relative surfactant content in the PBCA MB shell material.

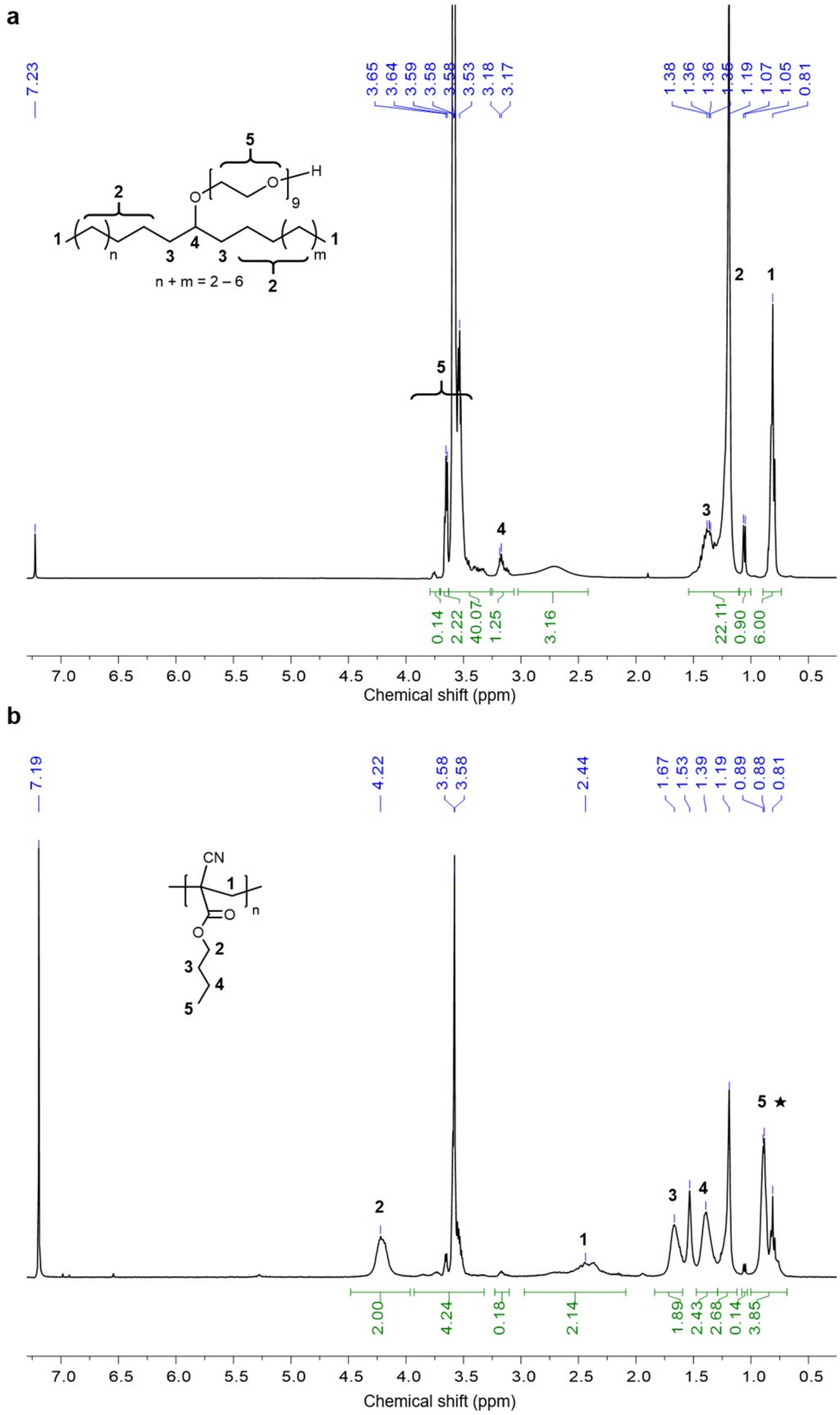

Figure S5. NMR spectra of Tergitol 15-S-9 and 15-S-9 MB. Spectra of (**a**) Tergitol 15-S-9 and (**b**) the corresponding PBCA MB sample with peak assignments. The star indicates the signal used to quantify the relative surfactant content in the PBCA MB shell material.

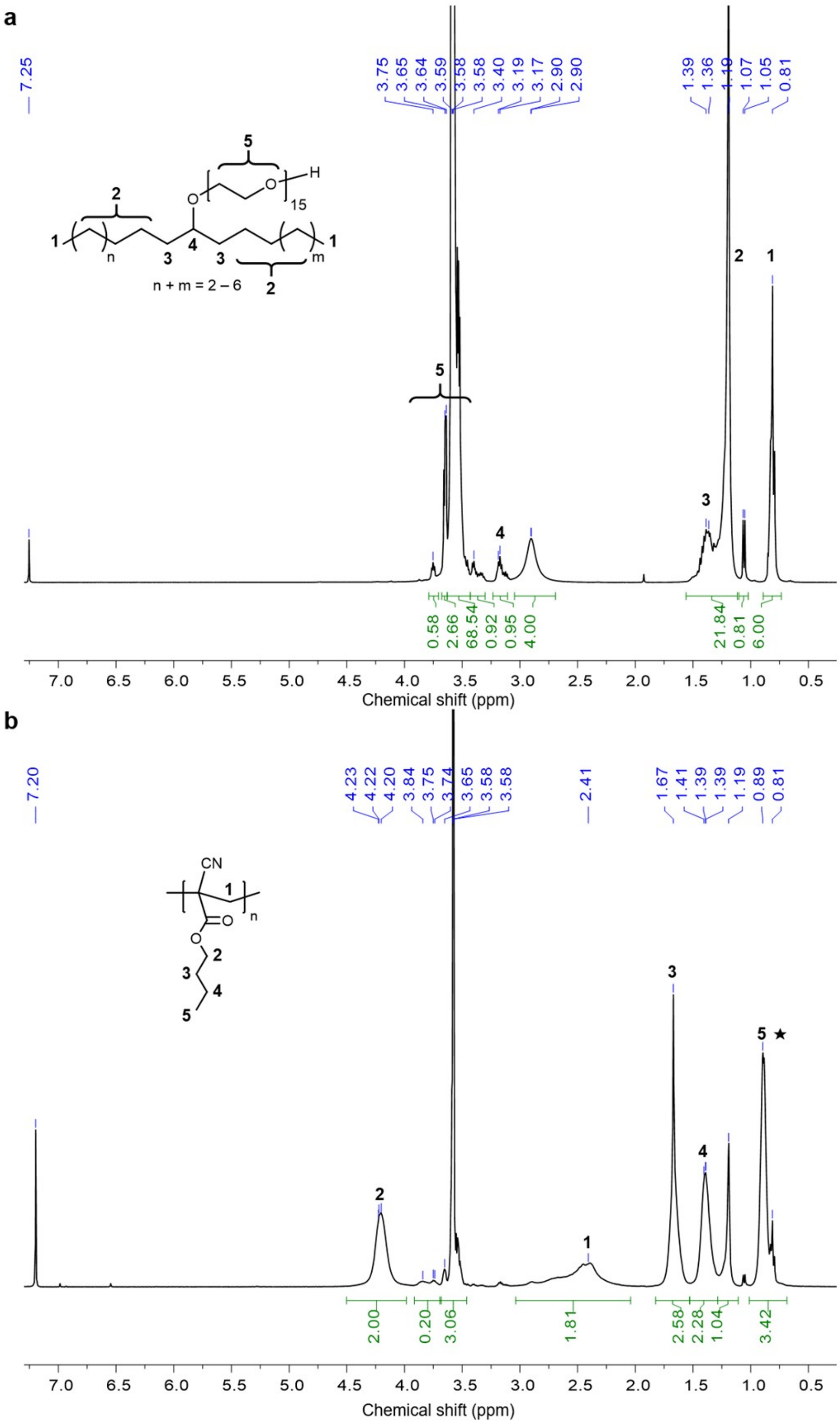

Figure S6. NMR spectra of Tergitol 15-S-15 and 15-S-15 MB. Spectra of (**a**) Tergitol 15-S-15 and (**b**) the corresponding PBCA MB sample with peak assignments. The star indicates the signal used to quantify the relative surfactant content in the PBCA MB shell material.

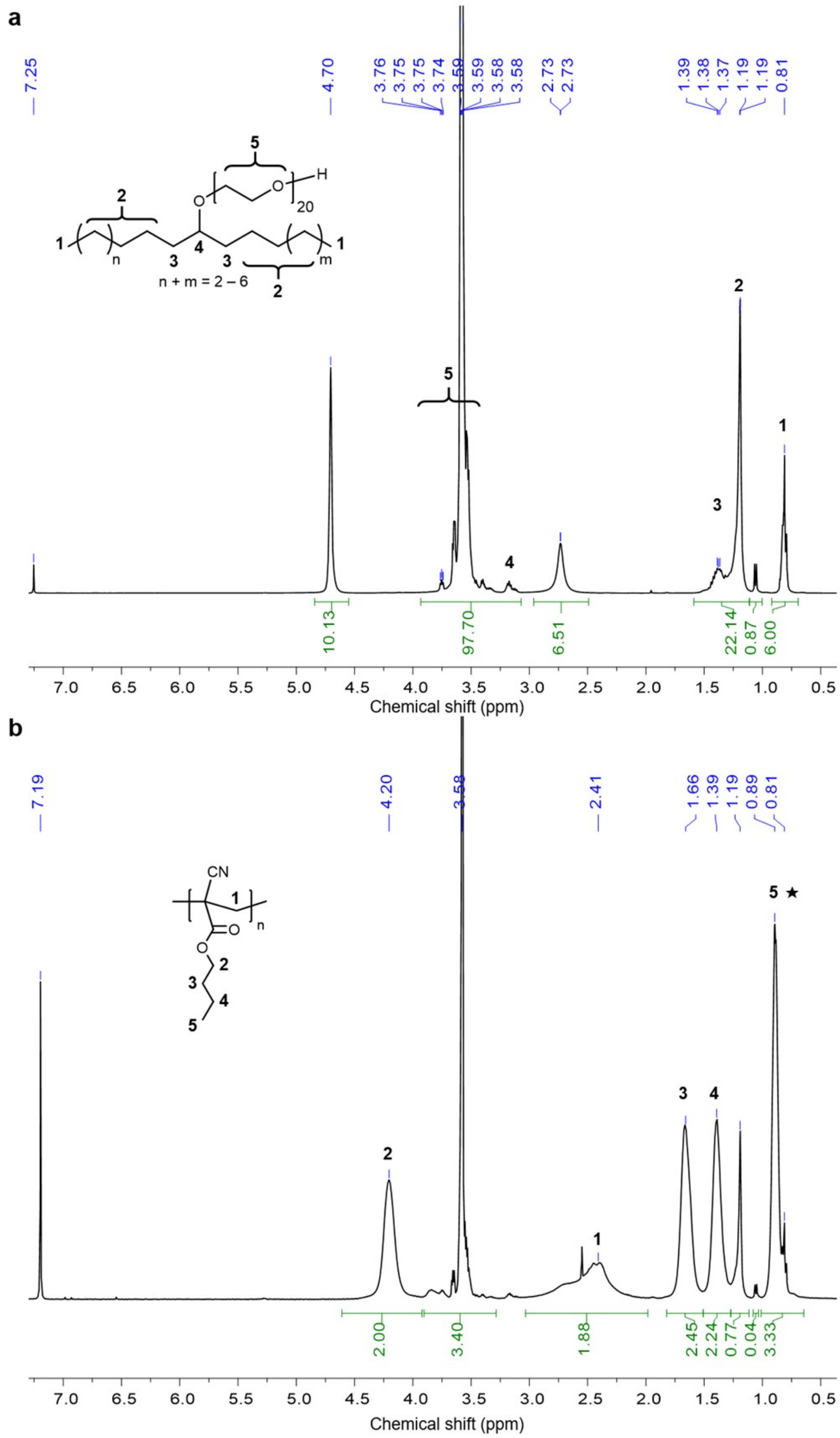

Figure S7. NMR spectra of Tergitol 15-S-20 and 15-S-20 MB. Spectra of (**a**) Tergitol 15-S-20 and (**b**) the corresponding PBCA MB sample with peak assignments. The star indicates the signal used to quantify the relative surfactant content in the PBCA MB shell material.

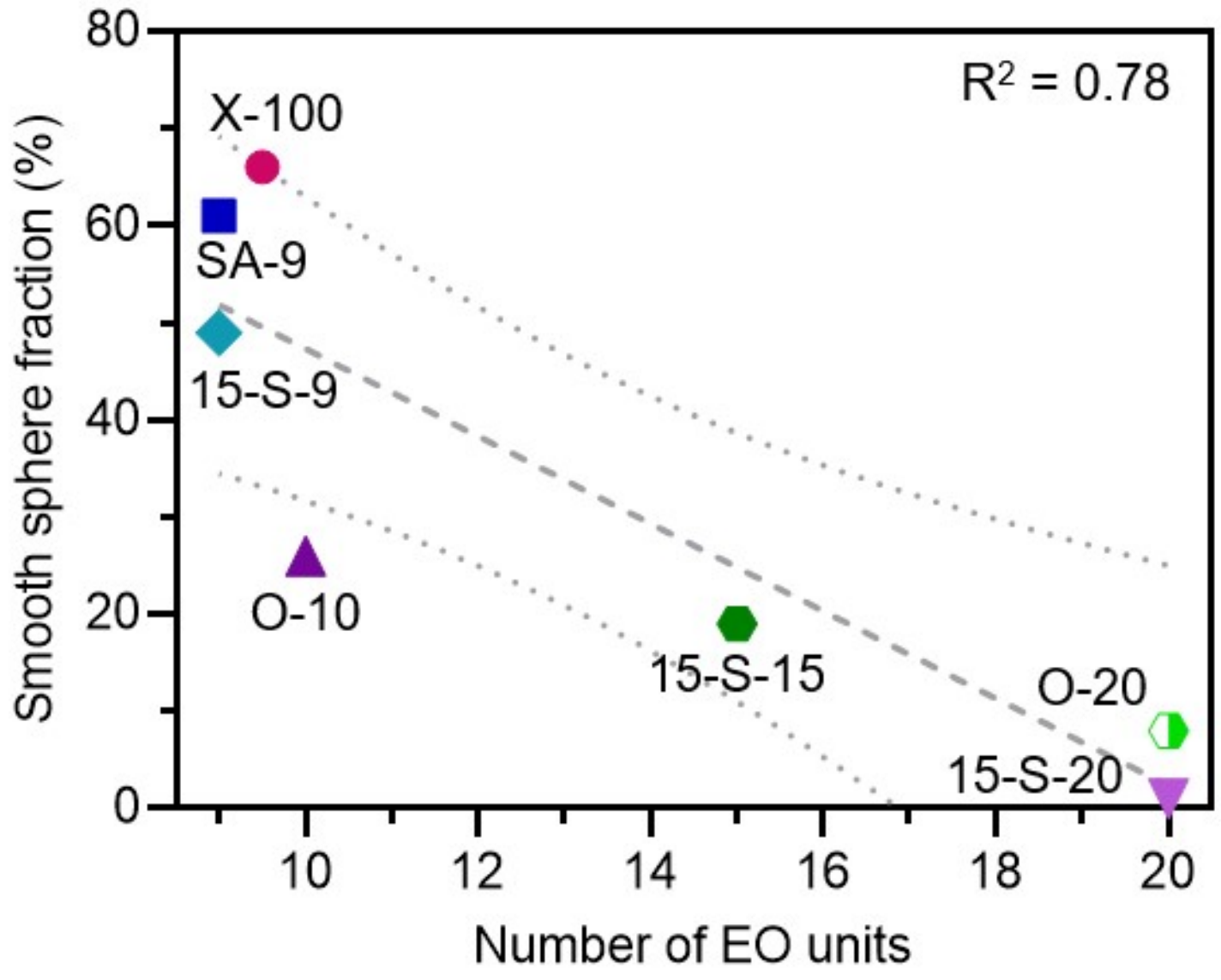

Figure S8. Correlation between surfactant EO number and the PBCA MB smooth sphere fraction. Gray dashed lines indicate the linear fit and its 95% confidence interval.

**Table S7.** Drug molecules per individual MB and drug loading per 1 × 10^9 MB.		
**Sample**	**Drug molecules per individual MB**	**Drug loading (μg / 1 × 10^9 MB)**
X-100 MB	(3.07 ± 0.65) × 10^6	1.79 ± 0.37
O-10 MB	(5.96 ± 1.13) × 10^6	3.47 ± 0.66
O-20 MB	(18.90 ± 5.32) × 10^6	11.00 ± 3.09
SA-9 MB	(14.74 ± 2.87) × 10^6	8.58 ± 1.67
15-S-9 MB	(1.03 ± 0.31) × 10^6	0.60 ± 0.18
15-S-15 MB	(6.02 ± 1.18) × 10^6	3.50 ± 0.69
15-S-20 MB	(7.35 ± 1.32) × 10^6	4.28 ± 0.77

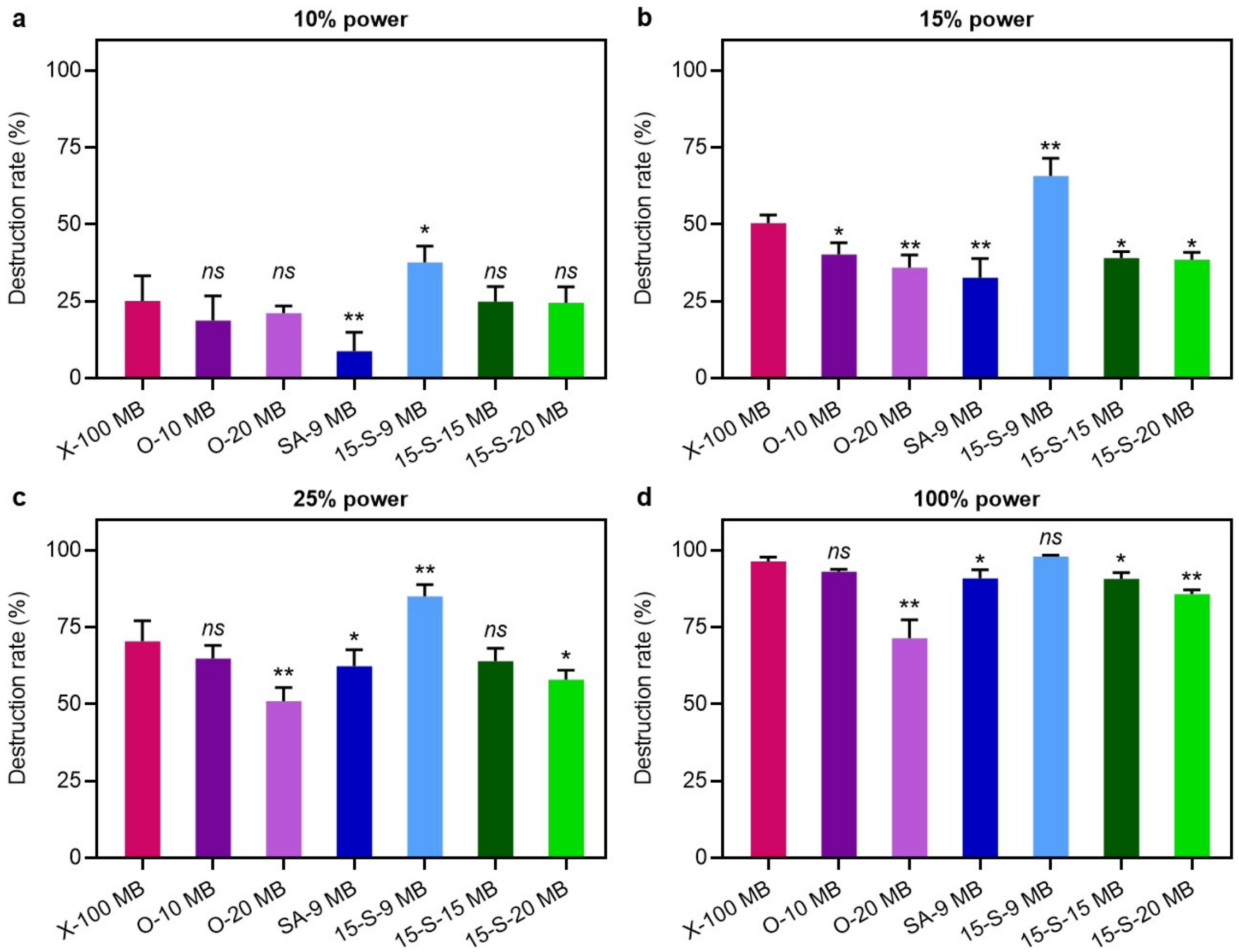

Figure S9. Destruction rates of the PBCA MB. (**a–d**) Destruction rate after exposure to (**a**) 10% power, (**b**) 15% power, (**c**) 25% power, and (**d**) 100% power for 5 s. (*) and (**) indicate groups that are significantly different with $p < 0.05$ and $p < 0.01$, respectively, while (*ns*) indicates groups that are not significantly different with $p > 0.05$ (one-way ANOVA with post hoc Tukey HSD test).

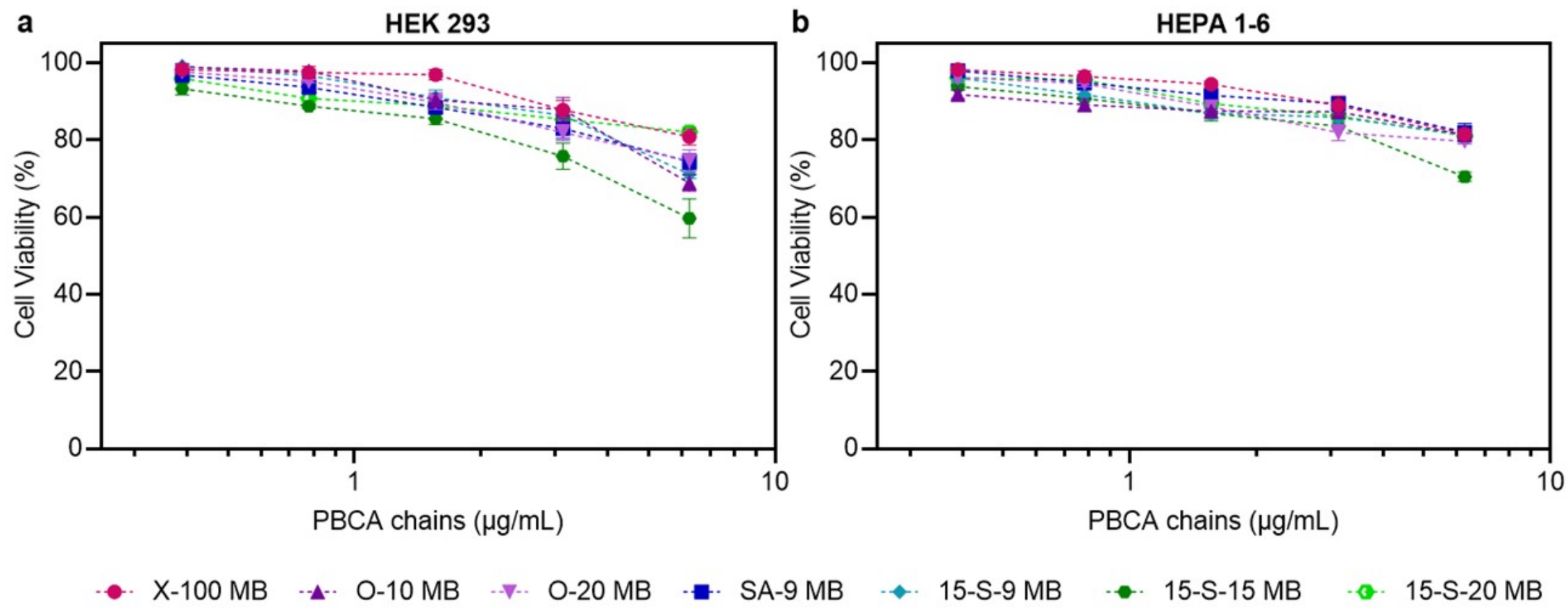

Figure S10. *In vitro* assessment of the PBCA chains. Cell viability of (**a**) HEK 293 cells and (**b**) Hepa 1–6 cells after 24 h incubations with corresponding PBCA MB shell materials.

REFERENCES

1 R. A. Barmin, A. Dasgupta, C. Bastard, L. De Laporte, S. Rütten, M. Weiler, F. Kiessling, T. Lammers and R. M. Pallares, *Mol. Pharm.*, 2022, **19**, 3256–3266.

2 M. Moosavifar, R. A. Barmin, E. Rama, A. Rix, R. A. Gumerov, T. Lisson, C. Bastard, S. Rütten, N. Avraham-Radermacher, J. Koehler, M. Pohl, V. Kulkarni, J. Baier, S. Koletnik, R. Zhang, A. Dasgupta, A. Motta, M. Weiler, I. I. Potemkin, G. Schmitz, F. Kiessling, T. Lammers and R. M. Pallares, *Adv. Sci.*, 2024, **11**, 2404385.

3 P. J. Hoogerbrugge and J. M. V. A. Koelman, *EPL (Europhysics Lett.*, 1992, **19**, 155–160.

4 P. Espanol and P. Warren, *EPL (Europhysics Lett.*, 1995, **30**, 191–196.

5 R. D. Groot and P. B. Warren, *J. Chem. Phys.*, 1997, **107**, 4423–4435.

6 R. D. Groot and K. L. Rabone, *Biophys. J.*, 2001, **81**, 725–736.

7 C. Hansen, *Hansen Solubility Parameters: A User's Handbook, Second Edition*, 2012.

8 T. Lindvig, M. L. Michelsen and G. M. Kontogeorgis, *Fluid Phase Equilib.*, 2002, **203**, 247–260.

9 D. W. Van Krevelen and K. Te Nijenhuis, in *Properties of Polymers (Fourth Edition)*, eds. D. W. Van Krevelen and K. B. T.-P. of P. (Fourth E. Te Nijenhuis, Elsevier, Amsterdam, 2009, pp. 189–227.

10 C. R. Iacovella, M. A. Horsch, Z. Zhang and S. C. Glotzer, *Langmuir*, 2005, **21**, 9488–9494.

11 Y. Li, M. Kröger and W. K. Liu, *Nanoscale*, 2015, **7**, 16631–16646.

12 A. P. Thompson, H. M. Aktulga, R. Berger, D. S. Bolintineanu, W. M. Brown, P. S. Crozier, P. J. in 't Veld, A. Kohlmeyer, S. G. Moore, T. D. Nguyen, R. Shan, M. J. Stevens, J. Tranchida, C. Trott and S. J. Plimpton, *Comput. Phys. Commun.*, 2022, **271**, 108171.

13 R. A. Barmin, M. Moosavifar, R. Zhang, S. Rütten, S. Thoröe-Boveleth, E. Rama, T. Ojha, F. Kiessling, T. Lammers and R. M. Pallares, *J. Mater. Chem. B*, 2024, **12**, 2511–2522.

14 P. S. Sheeran, Y. Daghighi, K. Yoo, R. Williams, E. Cherin, F. S. Foster and P. N. Burns, *Ultrasound Med. Biol.*, 2016, **42**, 795–807.

15 D. Wegierak, M. B. Cooley, R. Perera, W. J. Wulftange, U. A. Gurkan, M. C. Kolios and A. A. Exner, *IEEE Trans. Med. Imaging*, 2024, **43**, 2370–2380.
